# Uncovering the Pressure-Dependent Mechanism of CO_2_ Hydrogenation to Methanol on Ga-Promoted Cu/ZrO_2_ Using *Operando* Modulation-Excitation DRIFTS

**DOI:** 10.1021/jacs.5c04835

**Published:** 2025-07-25

**Authors:** Abdullah J. Al Abdulghani, Sudipta Ganguly, Ryan H. Hagmann, Zhuoran Sun, Matias Alvear, Lesli O. Mark, Eranda Nikolla, Yomaira J. Pagán-Torres, Ive Hermans

**Affiliations:** † Department of Chemical and Biological Engineering, 5228 University of Wisconsin−Madison, Madison, Wisconsin 53706, United States; ‡ Department of Chemistry, University of Wisconsin−Madison, Madison, Wisconsin 53706, United States; § Department of Chemical Engineering, 1259 University of Michigan, Ann Arbor, Michigan 48109, United States; ∥ Department of Chemical Engineering, 16146 University of Puerto Rico−Mayagüez, Mayagüez, Puerto Rico 00681, United States; ⊥ Wisconsin Energy Institute, University of Wisconsin−Madison, Madison, Wisconsin 53726, United States

## Abstract

The synthesis of methanol via CO_2_ hydrogenation is attracting significant interest, with Cu-based catalysts currently leading this promising approach. Incorporating Ga and Zr promoters further enhances catalyst performance by suppressing the competing reverse water–gas shift (RWGS) reaction. However, their precise mechanistic roles and the identities of key reaction intermediates remain debated, which may be the key for catalyst design and process optimization. In this study, we extend *operando* modulation-excitation spectroscopy coupled with diffuse reflectance infrared Fourier transform spectroscopy and mass spectrometry (ME-DRIFTS-MS) to investigate CO_2_ hydrogenation over Ga-promoted Cu/ZrO_2_ under varying industrially relevant pressures up to 50 bar. Our results indicate that methanol formation proceeds predominately via the formate pathway with formate (HCOO*) and methoxy (CH_3_O*) as pivotal intermediates. Additionally, we demonstrate that the rate-determining step is strongly dependent on the pressure and temperature, ultimately dictated by the local abundance of adsorbed hydrogen (H*) and gaseous H_2_O. Ga facilitates hydrogen adsorption, accelerating HCOO* hydrogenation to CH_3_O* and preventing its decomposition to CO. Notably, CH_3_O* conversion to CH_3_OH occurs via a water-assisted pathway rather than direct hydrogenation, explaining previously unclear correlation between Cu dispersion and catalytic activity. These mechanistic insights highlight the potential of optimizing reaction conditions—especially lower operating temperatures and controlled water cofeed—to significantly enhance methanol selectivity over Cu-based CO_2_ hydrogenation catalysts.

## Graphical abstract

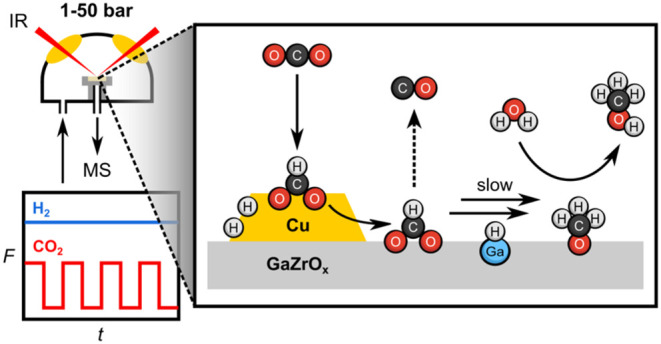

## 1 Introduction

The hydrogenation of CO_2_ to methanol has emerged as a potential route for CO_2_ utilization as an alternative carbon source.^1^ Methanol is an important commodity chemical produced in excess of 110 million tonnes per year.^2,3^ Most of the methanol produced today is from syngas originating from steam reforming of hydrocarbons.^4^ As green hydrogen is projected to be more available, pilot plants are constructed for CO_2_ hydrogenation. The largest of these plants was inaugurated in 2023 in Anyang, Henan province, China with a methanol production capacity of 110,000 tonnes per year.^5^ This is, however, still an order-of-magnitude lower than the capacity of a typical fossil fuel-based methanol production plant (reaching 1.8 million tonnes per year).^6,7^ The development of more efficient processes may help incentivize green methanol production.^8^

Methanol production from syngas (a mixture of CO and H_2_ with small amounts of CO_2_) is catalyzed by Cu-based materials (eq 1).^9,10^ The state-of-the-art Cu/ZnO/Al_2_O_3_ system, which has been developed over decades for syngas-to-methanol, is also active for CO_2_-to-methanol (CTM, eq 2).^9^ However, it also catalyzes the reverse water–gas shift reaction (RWGS, eq 3) that produces CO. To thermodynamically favor CTM over RWGS, the reactor needs to operate at high pressures and low temperatures (typically 30–100 bar and 200–300 °C, respectively).^11–13^

1 CO+2H2→CH3OHΔH°(25C°)=−94.5kJ mol−1

2 CO2+3H2⇌CH3OH+H2OΔH°(25C°)=−53.3kJ mol−1

3 $$\begin{array}{l} {\mathrm{CO}}_{2}+H_{2}⇌\mathrm{CO}+H_{2}O\\ \Delta H^{{}^{\circ}}\left(25^{{}^{\circ}}C\right)=+41.2\;{\mathrm{kJ mol}}^{-1} \end{array}$$

An interesting observation has been reported for In-based CO_2_ hydrogenation catalysts. Typically, these catalysts require higher operating temperatures as compared to Cu-based counterparts, to generate oxygen vacancies and partially reduced In_2_O_3–*x*_ active sites.^14,15^ Even at 300 °C where RWGS is thermodynamically favorable, In_2_O_3_/ZrO_2_ remarkably delivers a methanol selectivity close to 100% at 50 bar, H_2_/CO_2_ ratio of 4, and gas hourly space velocity (GHSV) of 16,000 h^–1^.^16^ In contrast, the selectivity toward methanol on Cu/ZnO/Al_2_O_3_ at steady state under the same conditions is only 11% at a lower CO_2_ conversion.^16^ This behavior implies that the pathway to produce CO through RWGS is kinetically blocked on In_2_O_3_/ZrO_2_, which poses the question whether Cu-based catalysts can be tuned to achieve higher selectivity by similarly blocking the pathway toward RWGS.

We have shown recently that the addition of Ga to Cu/ZrO_2_ raises the apparent barrier for CO formation from 89 to 113 kJ mol^–1^ while keeping the methanol formation barrier nearly constant at around 38 kJ mol^–1^.^17^ This change in the apparent barrier to form CO raises the methanol selectivity from 51% to 60% at an isoconversion of 7%.^17^ The Ga promotion illustrates the tunability of Cu-based materials, as was reported by other independent groups.^18–22^ We attributed this effect by Ga in CuGaZrO_*x*_ catalysts to the synergy between Ga and Cu to adsorb H, which facilitates the conversion of intermediates to methanol, rather than their irreversible decomposition to CO. In general agreement, we have shown that the observed methanol space-time-yield (STY) correlates significantly better with the H_2_/D_2_ exchange rate than the widely used Cu dispersion.^16^ Interestingly, we still found Zr to be needed for the highest methanol STY, playing its promotional role even in the presence of Ga.^23–28^ In this work, we aim to investigate the underlying mechanism of CO_2_ hydrogenation on CuGaZrO_*x*_ and the promotional roles using *operando* vibrational spectroscopy.

The pathways of methanol formation from syngas and CO_2_ over Cu-based catalysts have been previously investigated using reactivity studies, spectroscopy, and microscopy.^29–31^ A key question to understand methanol selectivity is how the CTM and RWGS reactions proceed. Through kinetic isotope effect (KIE) experiments on Cu/ZnO/Al_2_O_3_, Schlögl, Behrens, and co-workers concluded that the two reactions likely proceed through different pathways with no shared intermediates.^32^ While the mechanism of CO_2_ hydrogenation to methanol remains highly contentious, the observation by Schlögl, Behrens, and co-workers is in general agreement with the formate mechanism being the pathway for methanol production, whereas the carboxyl and/or direct CO_2_ dissociation mechanisms lead to CO formation (Scheme 1).^33^

**Scheme 1:**
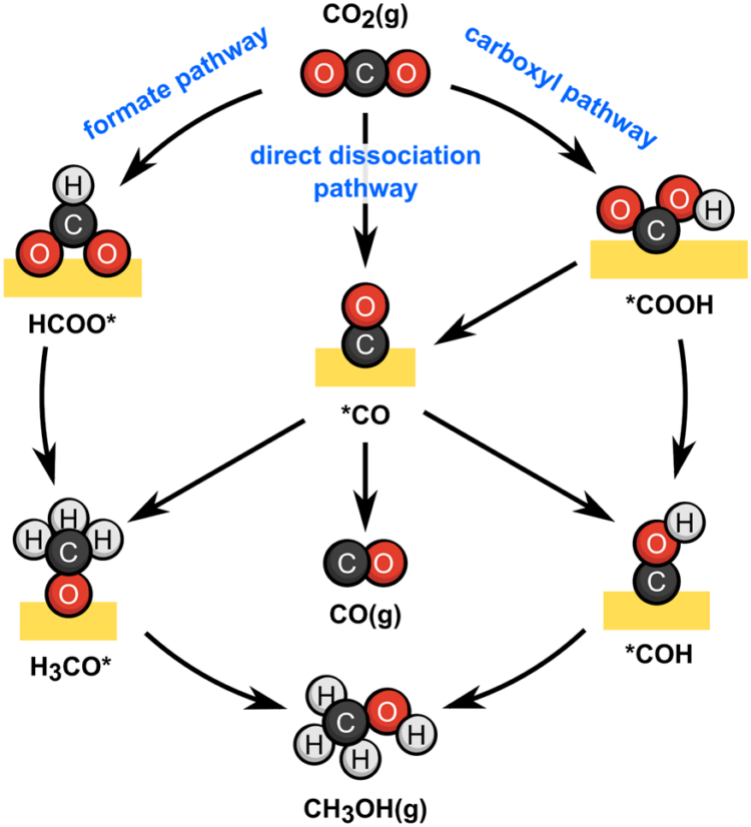
Simplified Pathways for CO_2_ Hydrogenation to Methanol and CO^a^

a An asterisk (*) denotes an adsorbed species

Formate (HCOO*) and methoxy (CH_3_O*) are the most reported intermediates for CO_2_ hydrogenation over Cu-based catalysts.^30^ Accordingly, their relevance to the catalytic cycle has been presumed in experimental mechanistic studies and was also justified by density functional theory (DFT) investigations.^28,29,33–37^ Mims and co-workers challenged this view by showing that the formate species on a Cu/SiO_2_ catalyst pre-exposed to formic acid did not generate methanol under 6 bar D_2_.^38^ However, the same catalyst showed methanol productivity under a D_2_/CO_2_ feed at the same partial pressure of D_2_. In a follow-up paper, Mims and co-workers showed with more transient experiments that formates are likely spectators or “dead ends” in the catalytic cycle and not active intermediates during the transformation of CO_2_ to methanol.^39^ Indeed, not all species observed by vibrational spectroscopy under reaction conditions are part of the catalytic cycles leading to observable products.^31,40–45^

To investigate the CO_2_ hydrogenation mechanism over CuGaZrO_*x*_ while addressing this challenge, we couple high-pressure diffuse reflectance infrared Fourier transform spectroscopy (DRIFTS), mass spectrometry (MS), and modulation-excitation spectroscopy (MES) in an *operando* manner, advancing the approach introduced by Maeda, Meier, and co-workers.^37^ In typical ME-DRIFTS experiments, periodic concentration perturbations are applied by switching the inlet feed to the DRIFTS cell between two feeds at a set frequency. As the system reaches a quasi-steady state, DRIFTS spectra of several modulation cycles are averaged as one cycle. Then, the phase sensitive detection (PSD) analysis is applied to keep only the signals that respond at the same frequency as the input perturbation according to (eq 4)­

4 $$A_{i,k}\left(\phi_{k}^{\mathrm{PSD}}\right)=\frac{2}{T}\int_{0}^{T}A_{i}\left(t\right)\sin\left(k\omega t+\phi_{k}^{\mathrm{PSD}}\right)dt$$

where *A*_*i,k*_(ϕ_*k*_^PSD^) and *A*_*i*_(*t*) are the responses in the phase (0 ≤ ϕ_*k*_^PSD^ ≤ 2π) and time (0 ≤ *t* ≤ *T*) domains, respectively, *i* is the spectral position (i.e., wavenumber in this study), *T* is the time period of one cycle, ω is the modulation frequency (equals 2π/*T*), and *k* is the demodulation index.^40–44^ The MES-PSD methodology significantly eliminates random noise and spectator signals, as neither closely follows the periodic perturbation at quasi-steady state.^40–44^ Moreover, the PSD analysis assigns each signal a phase delay, allowing the prediction of mechanisms and estimation of relative formation rates.^40–44^

When we switch the CO_2_ feed on and off periodically, our ME-DRIFTS-MS results indicate that HCOO* and CH_3_O* are indeed key intermediates during CO_2_ hydrogenation to methanol on CuGaZrO_*x*_, in favor of the formate pathway as the dominant mechanism. We find the rate-determining step to be a function of the operating pressure and temperature, which is ultimately related to the abundance of H* and H_2_O­(g) near the intermediates. HCOO* is formed on Cu and then stabilized by the metal oxide support. H* species on Ga seem to play a role in converting HCOO* to CH_3_O*. Finally, H_2_O­(g) hydrolyzes CH_3_O* to CH_3_OH­(g). Our findings not only uncover the critical role of Ga in improving the methanol selectivity, but also open up opportunities in engineering future CO_2_ hydrogenation catalysts and processes.

## 2 Results and Discussion

### 2.1 HCOO* as a Key Intermediate in CO_2_ Hydrogenation to Methanol

Coprecipitated Cu-GaZrO_*x*_-*Z* samples containing ∼20 wt% Cu and varying loads of Ga and Zr were synthesized as previously reported (refer to Tables S1 and S2 for composition, surface area, Cu dispersion, and reactivity).^17^ The *Z* in Cu-GaZrO_*x*_-*Z* refers to the percentage of the molar ratio Zr/(Zr + Cu + Ga). ME-DRIFTS-MS experiments were performed on these samples according to Table 1. The resulting phase-resolved DRIFTS spectra are shown in Figures S7–S37. In these experiments, the CO_2_ feed was switched on and off periodically while keeping that of H_2_ constant. The MS profiles during the MES experiment at 20 bar and 260 °C on Cu-GaZrO_*x*_-24 (sample with highest methanol STY and selectivity among tested CuGaZrO_*x*_, Table S2)^17^ show the successful modulation of CO_2_ and the periodic formation of the expected products—methanol, CO, and water (Figure 1a). The associated phase-resolved DRIFTS spectra (Figure 1b,c) show additionally the formation and modulation of HCOO*, CH_3_O* (Zr–OCH_3_, reference spectra in Figure S5), Ga–H, and multiple C–H stretching peaks. Their response to the periodic modulation at the same frequency indicates that HCOO* (1600 cm^–1^) and CH_3_O* (1152 cm^–1^) are indeed key intermediates in CO_2_ hydrogenation,^37^ hinting that methanol is likely formed through the formate pathway (CO_2_ → HCOO* → CH_3_O* → CH_3_OH, Scheme 1).

**Figure 1:**
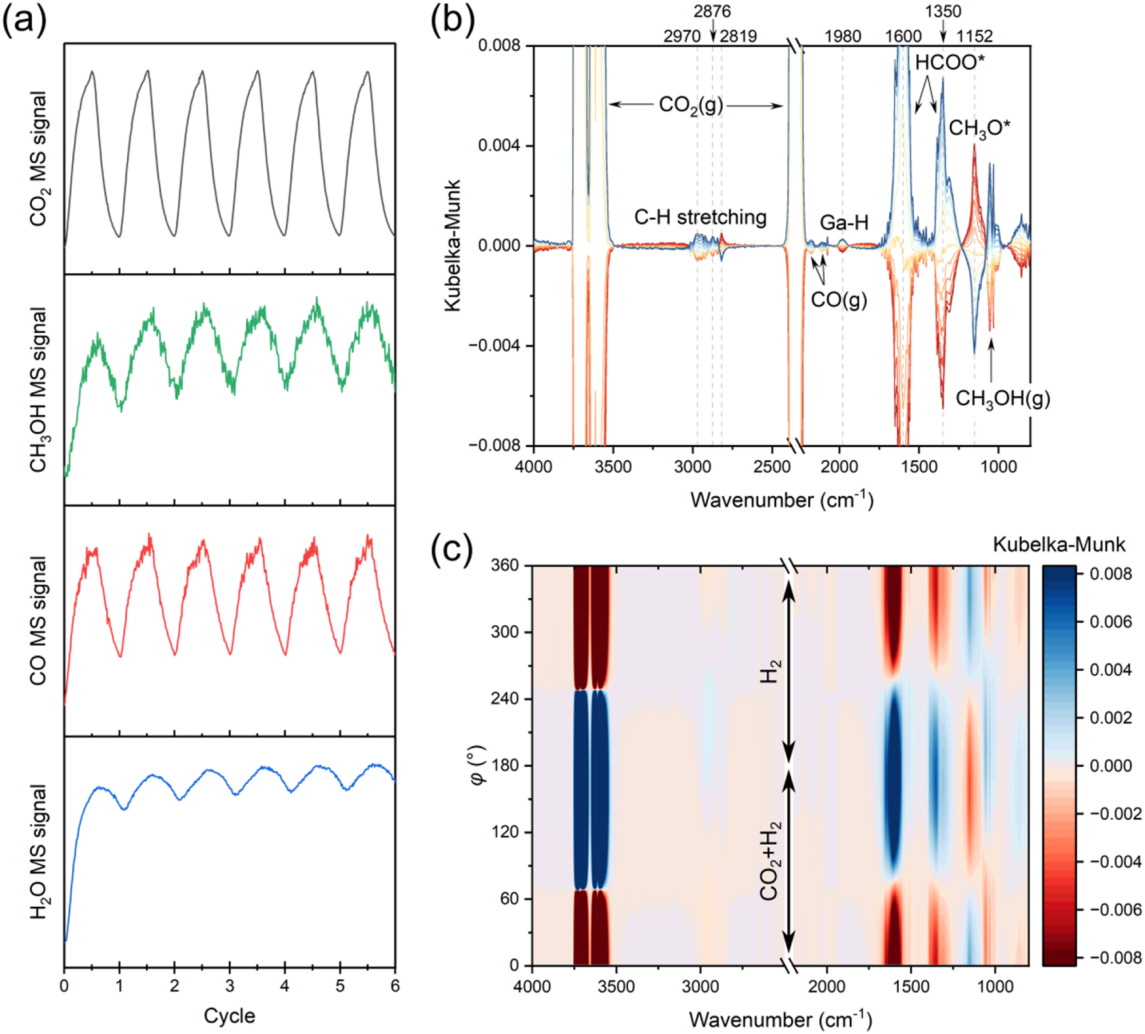
(a) MS profiles during the CO_2_ hydrogenation MES experiments on Cu-GaZrO_*x*_-24 at 20 bar and 260 °C using (*m*/*z* 44) for CO_2_, (*m*/*z* 31) for methanol, (*m*/*z* 28 corrected by subtracting the contribution from CO_2_) for CO, and (*m*/*z* 18) for H_2_O. (b) Phase-resolved DRIFTS spectra from 0° (red spectra, out-of-phase with CO_2_) to 180° (blue spectra, in-phase with CO_2_) with 15° increments. (c) Contour representation of the phase-resolved DRIFTS spectra. Refer to Table 1 for conditions.

**Table 1:** Implemented Partial Pressures, Total Flow Rates, and Time Periods at the Different Working Pressures^a^

*P*_total_ (bar)	*p*_H2_ (bar)	*p*_CO2_ (bar)	*F*_total_ (sccm)	*T* (min)
1	0.8	0.2	20	6
20	9.6	2.4	62.5	12
35	18.7	4.7	75	24
50	27.5	6.9	80	48

a Refer to the Supporting Information for estimation of dead volumes and times

Although our ME-DRIFTS-MS experiments indicate that HCOO* is part of the catalytic cycle during CO_2_ hydrogenation, it does not exclusively pinpoint whether the final product of HCOO* is methanol, CO, both, or neither. To demonstrate HCOO* association with methanol formation, we couple our ME-DRIFTS-MS findings with transient DRIFTS experiments. Inspired by the fact that a catalyst generally facilitates both the forward and reverse reactions, we performed the reverse reaction of CTM—the steam reforming of methanol (eq 5) on the CuGaZrO_*x*_ samples

5 $$\begin{array}{l} {\mathrm{CH}}_{3}\mathrm{OH}+H_{2}O⇌{\mathrm{CO}}_{2}+3H_{2}\\ \Delta H^{{}^{\circ}}\left(25^{{}^{\circ}}C\right)=+53.3\;{\mathrm{kJ mol}}^{-1} \end{array}$$

This reaction favors low pressures at equilibrium. Therefore, we carried it out at 1 bar and 260 °C (same temperature as our ME-DRIFTS-MS experiments). Samples were reduced *in situ* and then stabilized at 260 °C under pure Ar. Afterward, an Ar stream saturated with methanol at 10 °C was fed into the samples at 260 °C (Figure S2). The CO_2_ MS signal shows that the Cu-containing samples were able to convert methanol to CO_2_ (Figure 2). Since water was not fed into the samples, catalysts only performed this reaction during the initial first minutes, using residual water in the samples as verified by TGA-MS (Figure S38). Interestingly, the order of how well these samples catalyzed methanol steam reforming matched their reactivity order for CO_2_ hydrogenation to methanol: Cu/ZnO/Al_2_O_3_ > Cu-GaZrO_*x*_-24 > CuGaO_*x*_ > CuZrO_*x*_ ≫ GaZrO_*x*_ (Table S2).^17^ Although this observation may be a mere coincidence and the reactivity just matched the amount of stored water in the samples, it may rather suggest this reaction serves as a descriptor for the performance of CO_2_ hydrogenation catalysts and can be performed at a convenient pressure of 1 bar. The DRIFTS spectra of this experiment on Cu-GaZrO_*x*_-24 showed that HCOO* (1590 cm^–1^) was only observed when CO_2_(g) was being formed. While the forward and reverse reactions may follow different pathways, our experiments—motivated by microscopic reversibility—are macroscopically consistent with CO_2_ formation from methanol and water. We directly observed HCOO* as a surface intermediate in both directions, indicating its central role in CO_2_–methanol interconversion. After a short induction period, this particular sample started dehydrogenating methanol to formaldehyde. However, this transformation did not involve the peak at 1590 cm^–1^ that we assigned to HCOO*, further supporting that this peak is associated with a species containing 2 oxygen atoms (both methanol and formaldehyde contain 1 oxygen atom). Together from the ME-DRIFTS-MS and transient methanol steam reforming experiments, we conclude that the CO_2_ hydrogenation to methanol on CuGaZrO_*x*_ involves HCOO* as an intermediate.

**Figure 2:**
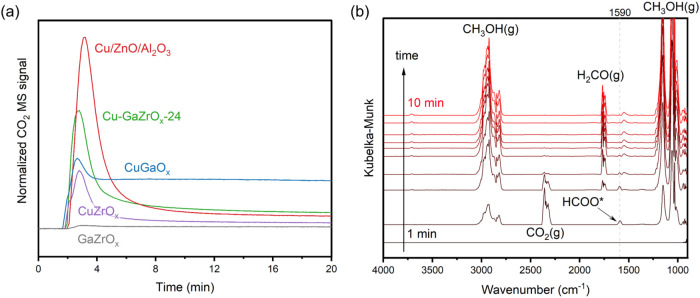
Methanol feed experiments at 1 bar and 260 °C over CuGaZrO_*x*_ catalysts. (a) Normalized CO_2_ MS signal (*I*_*m/z*44_/*I*_*m/z*40_). (b) Time-resolved DRIFTS spectra of the methanol feed experiment on Cu-GaZrO_*x*_-24.

### 2.2 Insignificant Contribution to Methanol Formation from CO Hydrogenation

Showing HCOO* is involved as an intermediate in CTM does not rule out any partial contribution from the other pathways (carboxyl and direct CO_2_ dissociation). In this section, we gauge the level of contributions from the other pathways to the formed methanol on CuGaZrO_*x*_. As shown in Scheme 1, *CO is a possible intermediate in both pathways. In fact, an important question that arises for CO_2_ hydrogenation catalysts is whether CO_2_ is hydrogenated directly to methanol (eq 2), or it is hydrogenated first to CO (eq 3) and then CO is hydrogenated to methanol (eq 1).^27,46^ This question is related to how selective a catalyst can be. If CO needs to form as an intermediate during CO_2_ hydrogenation to methanol over a catalyst, the maximum methanol selectivity this catalyst can achieve is determined by the thermodynamic equilibrium (∼30% at 300 °C and 50 bar using an inlet composition of 4:1 for H_2_/CO_2_,^47^ in contrast to the ∼100% methanol selectivity achievable experimentally on In_2_O_3_/ZnO_2_ at the same conditions).^16^ We address these questions by performing additional ME-DRIFTS-MS experiments on CuGaZrO_*x*_ by feeding and modulating CO instead of CO_2_.

We performed CO hydrogenation ME-DRIFTS-MS experiments at 20 bar and 260 °C on Cu-GaZrO_*x*_-24 using CO/H_2_/Ar as the feed. The MS profiles showed practically no methanol formation, especially from the third cycle (Figure 3). The slight methanol formation in the first cycle of the CO hydrogenation MES experiment could be attributed to CO­(g) turning to CO_2_(g) by forming oxygen vacancies in the metal oxides of Cu-GaZrO_*x*_-24. Then, CO_2_(g) was hydrogenated to methanol. The insignificant CO hydrogenation activity over Cu-GaZrO_*x*_-24 may stem from the low abundance of carbophilic Cu,^29^ due to the interaction between Cu and the Ga and Zr species.^17^ Ultimately, our data support that the primary source of carbon in methanol during CO_2_ hydrogenation is CO_2_, not CO, in the investigated pressure range. The inability of turning CO to methanol on CuGaZrO_*x*_ disfavors the carboxyl and direct CO_2_ dissociation as major pathways for methanol synthesis from CO_2_ hydrogenation.

**Figure 3:**
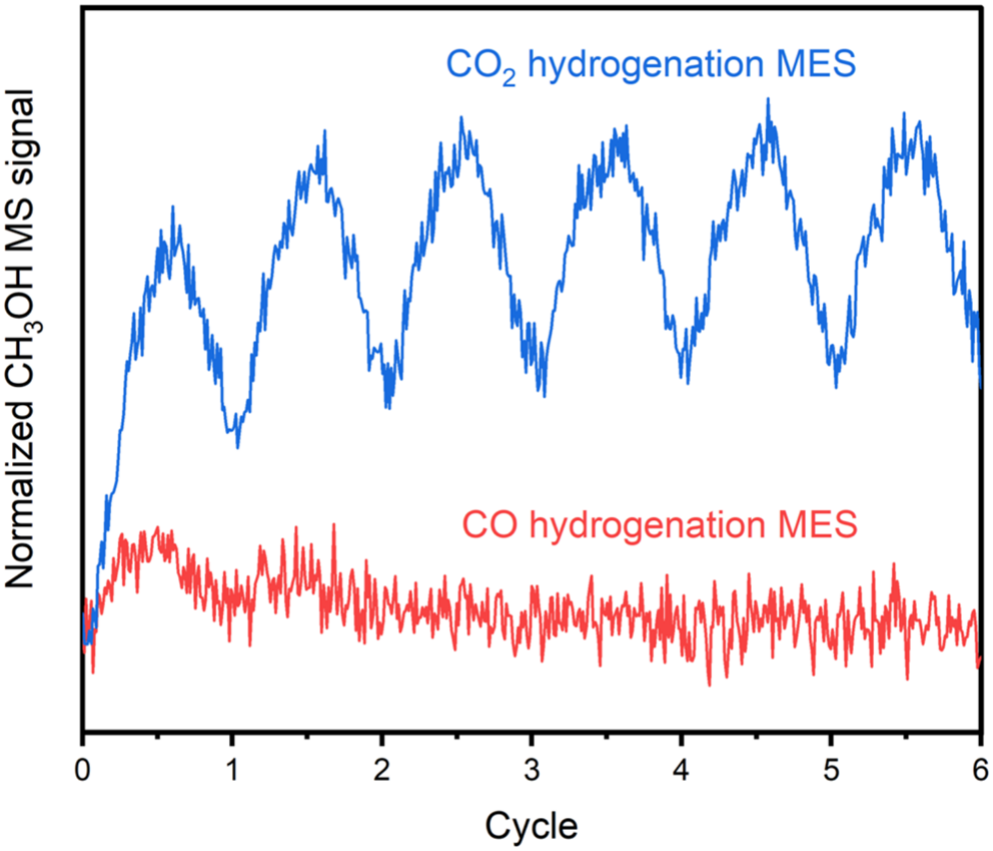
Normalized methanol MS signal (*I*_*m/z*31_/*I*_*m/z*2_) during CO_2_ and CO hydrogenation MES experiments on Cu-GaZrO_*x*_-24 at 20 bar and 260 °C.

### 2.3 Rapid Cu-Catalyzed HCOO* Formation

Now that we established that CO_2_ hydrogenation over CuGaZrO_*x*_ likely follows the formate pathway, we turn our investigation into its intermediates and their relative formation rates. The first intermediate of the formate pathway is HCOO*. During our ME-DRIFTS-MS experiments with CO_2_, the DRIFTS peaks associated with HCOO* appeared almost immediately after the introduction of CO_2_(g) (with a phase delay of 16° on average on all the Cu-containing samples at all tested temperatures and pressures, Table S5). HCOO* formation appears to be fast and unlikely to be the rate-determining step, in agreement with previous results,^37^ though we did not find enough evidence in our data supporting carbonates as an intermediate between CO_2_ and HCOO*. In fact, previous reports suggest formate formation from CO_2_ follows a fast Eley–Rideal mechanism (H* + CO_2_(g) → HCOO*) on Cu surfaces.^48,49^

As a control experiment, we performed ME-DRIFTS-MS on GaZrO_*x*_, which contains no Cu. At 20 bar, no methanol formation and relatively minor CO­(g) formation were detected by the MS (Figure 4a). In the associated phase-resolved DRIFTS spectra (Figure 4b), multiple peaks were formed during the CO_2_-rich half cycle, but neither of which peaked between 1590 and 1600 cm^–1^ which we assigned above to HCOO*. In fact, the in-phase peaks found on GaZrO_*x*_ from 1250 to 1700 cm^–1^ were found by just flowing CO_2_ on precipitated Ga_2_O_3_ and ZrO_2_ without H_2_ (Figure S3). Therefore, we assign these in-phase peaks (between 1250 and 1700 cm^–1^) on GaZrO_*x*_ as carbonates and bicarbonates, rather than HCOO*. There is, however, a hint of out-of-phase peaks in that region, along with an out-of-phase peak at 2876 cm^–1^, which we assign to HCOO*. In other words, CO_2_(g) turns into carbonates and bicarbonates on GaZrO_*x*_, and later in slow steps to HCOO*. These steps are likely catalyzed by Ga–H, peaking at 1976 cm^–1^ as reported in the literature^50–52^ and confirmed by our ME-DRIFTS-MS experiments using D_2_ instead of H_2_ (Figure 4c). However, in the time-resolved DRIFTS spectra at 20 bar (Figure 4d), the Ga–H peak appears to be largely static with only slight modulation of the peak during the MES experiments, hinting to its low ability to reduce the carbonates to HCOO* and/or the high stability of carbonates and bicarbonates. This observation highlights the importance of Cu in CuGaZrO_*x*_ for the rapid formation of HCOO*.

**Figure 4:**
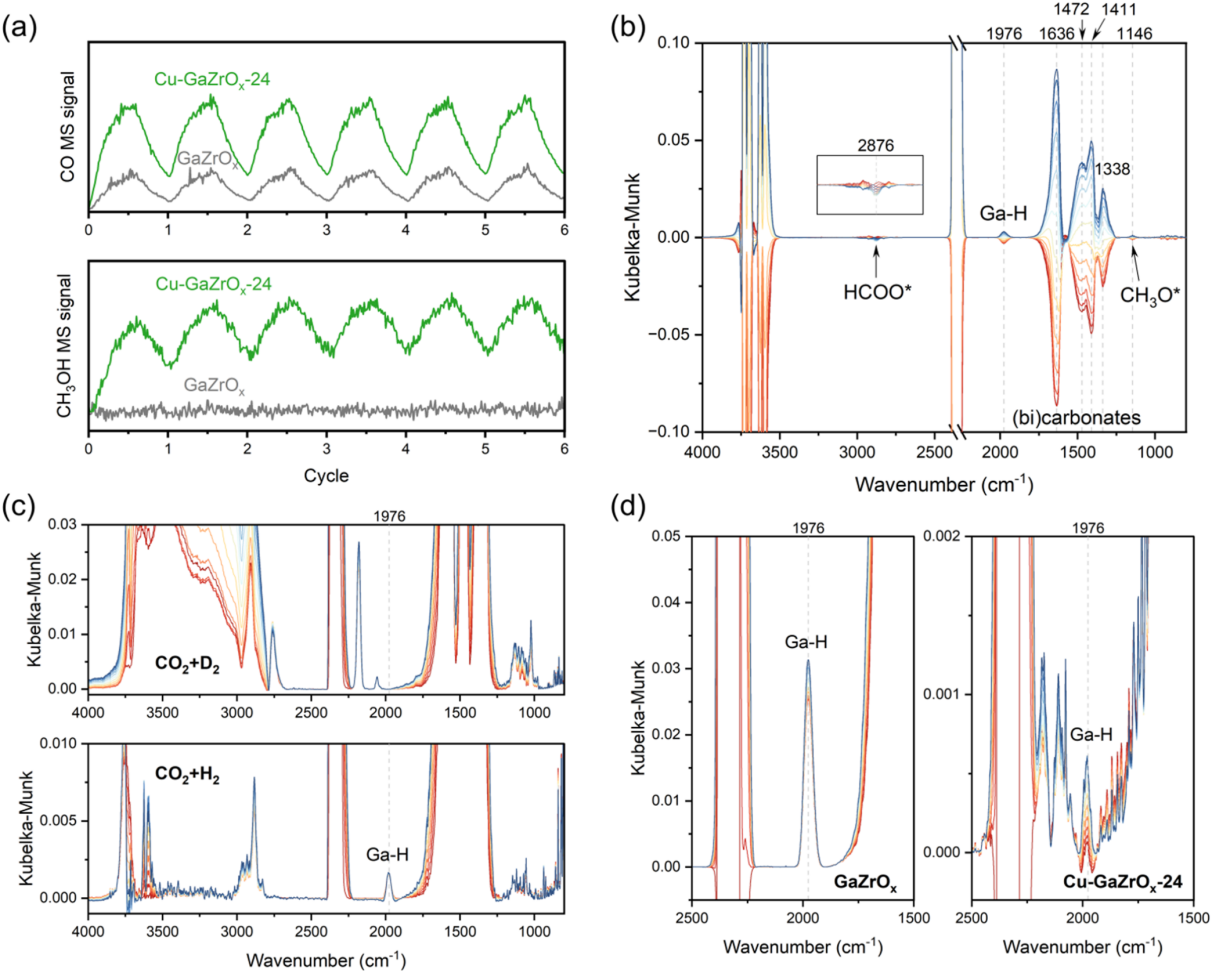
(a) Normalized MS response during MES experiments on Cu-GaZrO_*x*_-24 and GaZrO_*x*_ at 20 bar and 260 °C. (b) Phase-resolved DRIFTS spectra over GaZrO_*x*_ at the same conditions. (c) Time-resolved DRIFTS spectra of the MES experiments on GaZrO_*x*_ at 1 bar and 260 °C showing the effect of replacing H_2_ with D_2_ in the feed. (d) Time-resolved DRIFTS spectra of the MES experiments on GaZrO_*x*_ and Cu-GaZrO_*x*_-24 at 20 bar and 260 °C. All DRIFTS spectra are plotted from 0° to 180° with 15° increments.

### 2.4 Slow Transformation from HCOO* to CH_3_O*

In the literature, HCOO* hydrogenation and CH_3_O* conversion to methanol are both among the most proposed rate-determining steps for CO_2_ hydrogenation to methanol over Cu-based catalysts.^34–37,52–55^ An interesting observation from Figure 1b, as well as previously reported DRIFTS experiments,^37,55^ is the fact that CH_3_O* was out-of-phase, meaning that it peaked in a different half cycle than CO_2_(g). This behavior supports the argument that CH_3_O* formation from HCOO* is slower than CO_2_ hydrogenation to HCOO*. In other words, CO_2_ hydrogenation to HCOO* is fast during the CO_2_-rich half cycle. Because the hydrogenation of HCOO* to CH_3_O* is slow, the peak associated with CH_3_O* grows slowly from HCOO* and only reaches its maximum value during the half cycle without CO_2_(*g*). In agreement, performing an MES experiment on Cu-GaZrO_*x*_-24 at 20 bar and 260 °C (same sample and conditions as in Figure 1) and modulating H_2_ feed instead of CO_2_ resulted in an in-phase CH_3_O* (Figure S14c,d), since accumulated HCOO* cannot be hydrogenated to CH_3_O* in the half cycles without H_2_. This transformation involves multiple elementary steps that sum into the overall reaction shown in (eq 6)­

6 $${\mathrm{HCOO}}^{*}+4H^{*}\to H_{2}O+{\mathrm{CH}}_{3}O^{*}+4^{*}$$

Back to our default mode of modulating CO_2_ while keeping H_2_ constant, it is expected that HCOO* hydrogenation to CH_3_O* is faster at higher partial pressures of H_2_ due to the higher abundance of H* since (H_2_ + 2* → 2H*) is facilitated. The phase-resolved DRIFTS spectra of the MES experiments on Cu-GaZrO_*x*_-24 at varying pressures are shown in Figure 5a. Indeed, when the H_2_ partial pressure increased from 9.6 to 18.7 bar, the peak associated with CH_3_O* switched from being out-of-phase to being in-phase, peaking in the same half cycle as CO_2_(g). Additionally, given the reaction (H_2_ + 2* → 2H*) is likely exothermic,^33^ lowering the temperature (from 260 to 220 °C) is expected to result in higher H* concentrations (in line with H_2_-TPD results)^17^ that should accelerate HCOO* to CH_3_O* transformation (eq 6). Our results show that CH_3_O* also became in-phase by lowering the temperature at the same pressure of 20 bar (Figure 5a). In agreement, lowering the temperature also lowers the MS phase delay between methanol and CO_2_ signals and increases that between CO and CO_2_ (Figure S39a).

**Figure 5:**
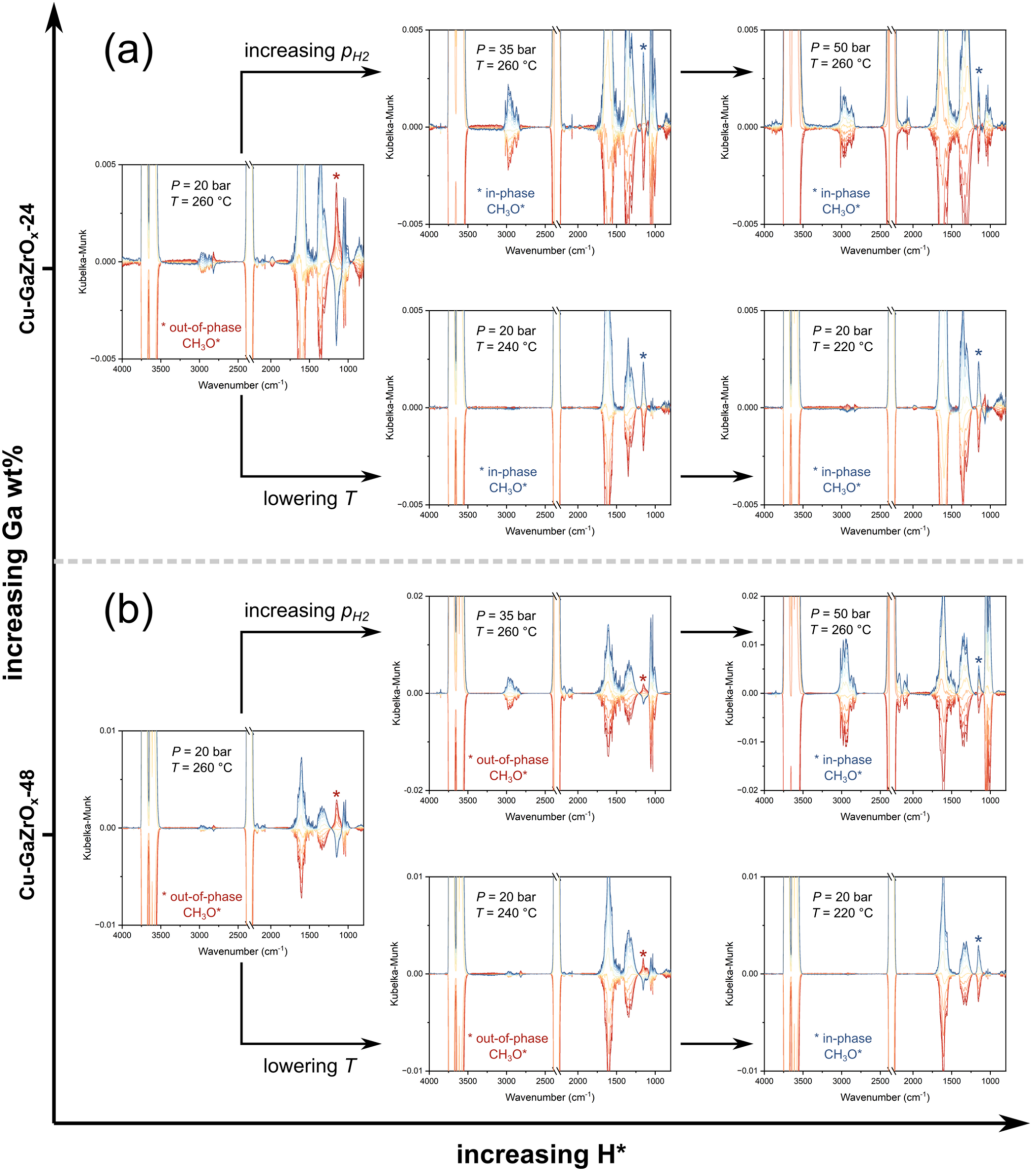
Effect of Ga content, H_2_ partial pressure, and temperature on the phase angle of CH_3_O* in the phase-resolved DRIFTS spectra of the CO_2_ hydrogenation MES experiments. DRIFTS spectra are plotted from 0° to 180° with 15° increments. Species evolution profiles as a function of phase angle are plotted in Figure S40.

We previously observed that as the Ga content increases in CuGaZrO_*x*_, more H is adsorbed and stabilized.^17^ We utilized this finding to examine our hypothesis that the transformation from HCOO* to CH_3_O* is slow and facilitated by the abundance of Ga–H. We thus examined the effect of lowering the Ga content. Over Cu-GaZrO_*x*_-24 (22 wt% Ga), a pressure of 35 bar was sufficient to make the CH_3_O* peak in-phase. However, the CH_3_O* peak was still out-of-phase over Cu-GaZrO_*x*_-48 (9 wt% Ga) at the same pressure, confirming that the lower abundance of H* on Cu-GaZrO_*x*_-48 slows down CH_3_O* formation. The same argument holds true by lowering the temperature at 20 bar (Figures 5b and S39b).

Considering the cases where the peak associated with CH_3_O* is in-phase, it may be possible that the peak also grows again in the other half cycle without CO_2_. That is because both CH_3_O* and HCOO* are saturated during the half cycle with CO_2_(g). When CO_2_(g) is switched off during the ME-DRIFTS-MS experiments, the concentration of both species starts to decay. If CH_3_O* decays faster than HCOO*, it may be possible to detect some CH_3_O* formation from HCOO* in the half cycle without CO_2_. This requires an inspection of the higher harmonics of the ME-DRIFTS-PSD spectra. Results from the fundamental frequency alone contain just one sinusoidal function for each wavenumber and therefore do not allow for a species to grow at different times within one cycle. Figure 6a shows the ME-DRIFTS-PSD spectra with higher harmonics of the CO_2_ hydrogenation experiment on CuGaZrO_*x*_-48 at 50 bar. Starting from the inclusion of up to *k* = 10, there is a clear growth of the CH_3_O* peak at the half cycle without CO_2_. This observation highlights the importance of inspecting the PSD higher harmonics, which are often neglected in MES-PSD catalysis studies,^37,56,57^ to detect complex dynamics such as the growth of one species at multiple times during one modulation cycle.

**Figure 6:**
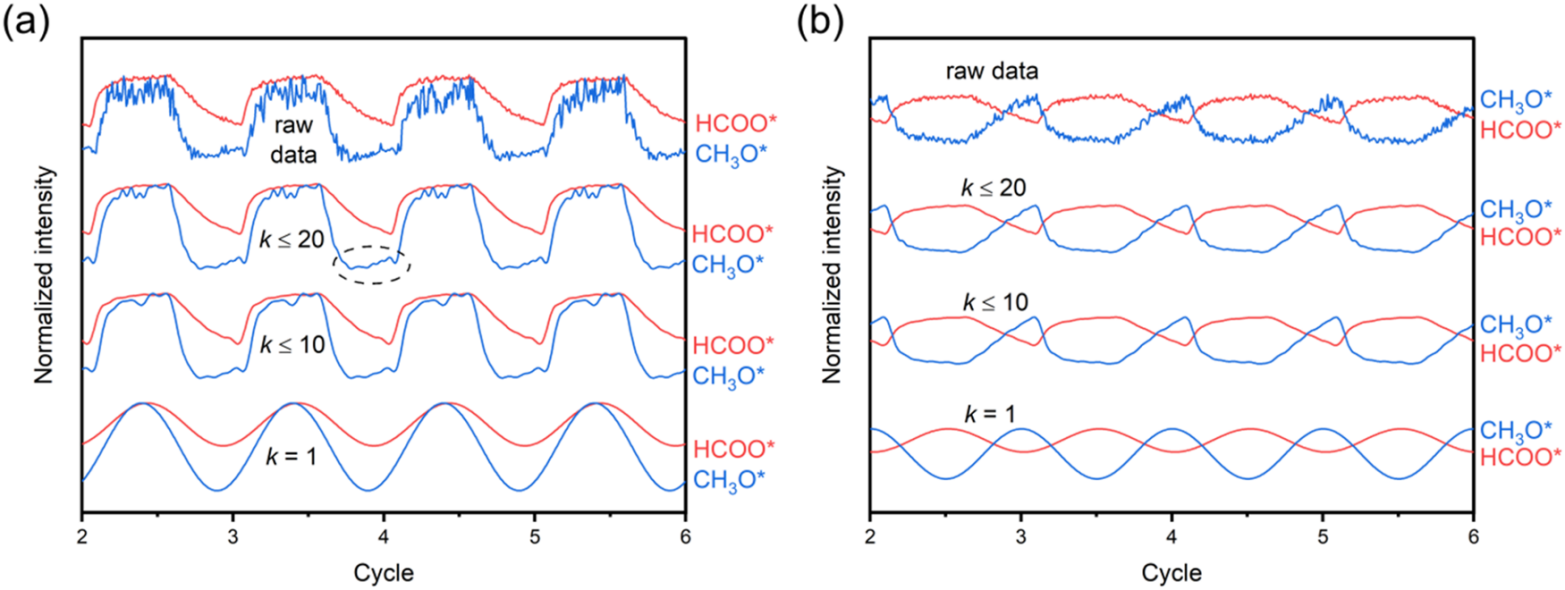
(a) Regrowth of the time-resolved CH_3_O* DRIFTS peak during the half cycle without CO_2_ in the MES experiment on Cu-GaZrO_*x*_-48 at 50 bar and 260 °C. The dashed ellipse highlights an example of such an instance. (b) Sawtooth-shaped profile of the time-resolved CH_3_O* DRIFTS peak in the MES experiment on Cu-GaZrO_*x*_-48 at 35 bar and 260 °C. *k* refers to the demodulation index of the PSD analysis.

### 2.5 Water-Catalyzed CH_3_O* Conversion to CH_3_OH­(g)

Finally, we examine the transformation from CH_3_O* to CH_3_OH­(g), which is the last step of the formate-mediated CO_2_ hydrogenation mechanism to methanol. This step becomes the rate-determining step at high pressures (≥35 bar) and also at low temperatures (220–240 °C). At 50 bar and 260 °C, the phase delay between CH_3_O* and CH_3_OH­(g) is 20° on Cu-GaZrO_*x*_-24 and 28° on Cu-GaZrO_*x*_-48, showing considerable delays between the surface species and the gaseous product. More importantly, in the conditions where CH_3_O* is out-of-phase, there is no evidence, from MS or DRIFTS, for CH_3_OH­(g) formation in the same half cycle as CH_3_O*. In other words, CH_3_OH­(g) seems to always form during the half cycle in which CO_2_ is fed. A hypothesis that could explain this behavior is that CH_3_O* is hydrolyzed to CH_3_OH­(g) (eq 7), as suggested by Fisher and Bell for Cu/ZrO_2_/SiO_2_,^53^ rather than its reduction by H* (eq 8)^33,37,55^

7 $${\mathrm{CH}}_{3}O^{*}+H_{2}O\left(g\right)\to {\mathrm{CH}}_{3}\mathrm{OH}\left(g\right)+{\mathrm{OH}}^{*}$$

8 $${\mathrm{CH}}_{3}O^{*}+H^{*}\to {\mathrm{CH}}_{3}\mathrm{OH}\left(g\right)+2^{*}$$

This distinction has been associated with the different charges of H species required to form C–H and O–H bonds.^14,15^ The H* species on metals are hydridic whereas the H atoms in water are protonic. Water is mostly formed during the half cycle with CO_2_ due to the RWGS (eq 3). When accumulated HCOO* slowly forms CH_3_O* in the half cycle without CO_2_, CH_3_O* keeps accumulating until the next half cycle when CO_2_(g) is fed again and freshly formed H_2_O­(g) rapidly hydrolyzes CH_3_O* to CH_3_OH­(g). This can also explain the unusual sawtooth shape of the CH_3_O* peak during the MES experiments (Figure 6b). This shape is an indication that CH_3_O* forms slowly, but gets consumed rapidly in the half cycle with CO_2_. It also indicates that CH_3_O* has a good stability on CuGaZrO_*x*_ and does not easily decompose to CO­(g), contrary to what was reported for this species on Cu-Zn-Zr-Ba/Al_2_O_3_.^37^ Since CO­(g) continues to form at the beginning of the half cycle without CO_2_, we hypothesize this CO­(g) formation comes from HCOO*, not CH_3_O* (Figure S41).

## 3 Conclusions

In this study, we spectroscopically investigated the mechanism of CO_2_ hydrogenation over CuGaZrO_*x*_ catalysts and the synergy between Cu, Ga, and Zr. The results from our ME-DRIFTS-MS experiments indicate that HCOO* and CH_3_O* are both key intermediates during CO_2_ hydrogenation, responding to the CO_2_ concentration perturbation at the same frequency. We further linked HCOO* to the pathway connecting CO_2_ to methanol by performing transient DRIFTS-MS experiments of methanol steam reforming over the samples. Limited methanol formation was observed during ME-DRIFTS-MS experiments of CO hydrogenation, instead of CO_2_ hydrogenation, allowing us to conclude that methanol is formed over CuGaZrO_*x*_ through the formate pathway. In this pathway, HCOO* is formed rapidly over Cu surfaces and then stabilized by the metal oxides. The transformations from HCOO* to CH_3_O* proceed through slow steps at low concentrations of H*, which is the case at low partial pressures of H_2_ and high temperatures. Increasing the Ga content in the CuGaZrO_*x*_ samples helps in facilitating HCOO* to CH_3_O* conversion, which we attribute to Ga stabilizing H* species near the surface intermediates. This mechanistic picture (Scheme 2) illustrates the promotional effect of Ga in methanol synthesis over CuGaZrO_*x*_, as the rapid conversion of HCOO* to CH_3_O* prevents its decomposition to CO. Furthermore, such a mechanistic picture provides a possible explanation to our previous observation that the methanol formation rates over CuGaZrO_*x*_ did not correlate with Cu dispersion. Given that HCOO* is formed rapidly on Cu surfaces and then stabilized by the metal oxides until its conversion to methanol in slow steps, the methanol formation rates are not expected to scale with Cu dispersion.

**Scheme 2:**
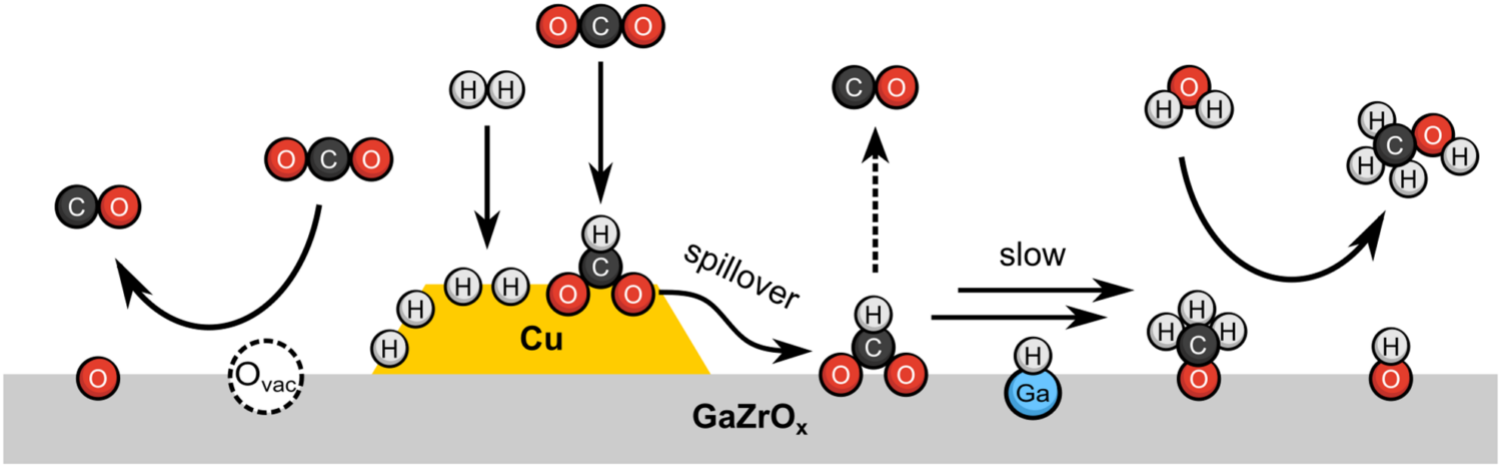
Proposed Mechanism of CO_2_ Hydrogenation on CuGaZrO_*x*_

Finally, we argue that CH_3_O* is likely hydrolyzed to CH_3_OH, rather than hydrogenated. This argument is supported by the fact that CH_3_OH­(g) appears to form only during the CO_2_-rich half cycles in the CO_2_ hydrogenation MES experiments. We show that CH_3_O* can be formed during the half cycles without CO_2_ from accumulated HCOO*, and the formed CH_3_O* gradually accumulates on the surface until it is rapidly transformed when CO_2_ is switched back on and water is produced. Due to the asymmetric formation and utilization rates of CH_3_O*, its DRIFTS signal exhibits a distinct sawtooth shape, which is encoded in the PSD higher harmonics. DFT studies have reported considerably lower activation energies for CH_3_O* conversion to CH_3_OH by water than by H* on Cu/ZrO_2_ and ZnO.^58^ Surface hydroxyls, which are expected to be more abundant in the presence of H_2_O, have also been reported to lower the barrier for the reductive conversion of CH_3_O* to methanol on Cu/ZnO.^54^

Experimentally, water has been reported to inhibit the methanol formation rates from CO_2_ hydrogenation over Cu/ZnO/Al_2_O_3_.^35,59,60^ We emphasize that CH_3_O* hydrolysis to CH_3_OH is only rate-determining when H* is abundant. A Cu-based catalyst that is rate-controlled by HCOO* conversion to CH_3_O* is not expected to benefit from cofeeding water because (i) CH_3_O* hydrolysis occurs after the rate-determining step, and (ii) water may inhibit H adsorption, which is required for the hydrogenation of HCOO* to CH_3_O*. It should be noted that not all Cu-based CO_2_ hydrogenation catalysts are the same. Li, Chen, Wang, and co-workers have demonstrated through isotope-tracing experiments that the addition of suitable amount of water in the feed improves the rate of CO_2_ hydrogenation to methanol over Cu-ZnO-ZrO_2_.^61^ Future work may assess the effect of cofeeding water at varying partial pressures of H_2_ over different Cu-based systems. Interestingly, forming CH_3_O* over CuGaZrO_*x*_ appears to be more facile at low temperatures (∼220 °C), which are not favorable conditions for RWGS. Therefore, a strategy to increase methanol selectivity may be to perform CO_2_ hydrogenation over CuGaZrO_*x*_ at low temperatures with additional water in the feed, opening up opportunities for more selective and efficient methanol synthesis processes from CO_2_ hydrogenation.

## Supplementary Material



## Notes

Detailed experimental methods, setup schematics, reference DRIFTS spectra, estimation of dead volumes, phase-resolved spectra of all MES experiments, tabulated phase delays, H_2_O-TPD profile, ME-DRIFTS intensity profiles, and effect of temperature on product formation (PDF)

## ∇

The Center for Energy Systems Design (CESD), International Institute for Carbon-Neutral Energy Research (WPI-I2CNER), Kyushu University, Fukuoka 819–0395, Japan

## Abbreviations

DRIFTS: diffuse reflectance infrared Fourier transform spectroscopy
MES: modulation-excitation spectroscopy
PSD: phase-sensitive detection
CTM: CO_2_ to methanol
RWGS: reverse water–gas shift
TGA: thermogravimetric analysis
MS: mass spectrometry
*m*/*z*: mass-to-charge ratio
GHSV: gas hourly space velocity
