# Supplementary material for: Uncovering the Pressure-Dependent Mechanism of CO2 Hydrogenation to Methanol on Ga-Promoted Cu/ZrO2 Using Operando Modulation-Excitation DRIFTS
Source: J Am Chem Soc. 2025 Jul 25;147(31):27438–48. doi: 10.1021/jacs.5c04835 (PMC12503360; doi:10.1021/jacs.5c04835)
Supplement: Supplementary file 1 [file ja5c04835_si_001.pdf]

Supporting information

Uncovering the pressure-dependent mechanism of CO_2 hydrogenation to methanol on Ga-promoted Cu/ZrO_2 using *operando* modulation-excitation DRIFTS

Abdullah J. Al Abdulghani,[a,b,‡] Sudipta Ganguly,[c,‡] Ryan H. Hagmann,[c] Zhuoran Sun,[c] Matias Alvear,[a] Lesli O. Mark,[a] Eranda Nikolla,[d] Yomaira J. Pagán-Torres,[e,*] Ive Hermans [a,c,f,*]

[a] Department of Chemical and Biological Engineering, University of Wisconsin–Madison, Madison, WI 53706, USA

[b] current address: The Center for Energy Systems Design (CESD), International Institute for Carbon-Neutral Energy Research (WPI-I2CNER), Kyushu University, Fukuoka 819-0395, Japan

[c] Department of Chemistry, University of Wisconsin–Madison, Madison, WI 53706, USA

[d] Department of Chemical Engineering, University of Michigan, Ann Arbor, MI 48109, USA

[e] Department of Chemical Engineering, University of Puerto Rico–Mayagüez, Mayagüez, PR 00681, USA

[f] Wisconsin Energy Institute, University of Wisconsin–Madison, Madison, WI, 53726, USA

‡ These authors contributed equally to this work.

* E-mail address: yomairaj.pagan@upr.edu (Y. J. Pagán-Torres); hermans@chem.wisc.edu (I. Hermans).

Table of Contents

1. Experimental methods S3

1.1. Catalysts S3

1.2. Experimental setup S3

1.3. ME-DRIFTS-MS experiments S4

1.4. MES-PSD analysis of DRIFTS data S5

1.5. Methanol steam reforming experiments S5

1.6. Reference DRIFTS spectra S5

1.7 Temperature-programmed desorption of water S5

2. Reference DRIFTS spectra on single components S6

2.1. (Bi)carbonates on ZrO_2 and Ga_2O_3 ...... S6

2.2. Formates on ZrO_2 and Ga_2O_3 ...... S7

2.3. Methoxy on ZrO_2 and Ga_2O_3 ...... S8

3. Estimation of dead volume and time S9

4. Phase-resolved DRIFTS spectra of CO_2 hydrogenation MES experiments on $CuGaZrO_x$ S10

4.1. Cu-GaO_x S10

4.2. Cu-$GaZrO_x$-24 ...... S13

4.3. Cu-$GaZrO_x$-48 ...... S16

4.4. Cu-ZrO_x S19

4.5. 10Cu-ZrO_x S22

4.6. $GaZrO_x$ S24

5. Supplementary figures and tables to the manuscript S27

6. References S32

1. Experimental methods

1.1. Catalysts

The $CuGaZrO_x$ samples were synthesized by coprecipitation as reported previously.[1] The metallic loadings, surface areas, and copper dispersion are summarized in **Table S1**. Results of reactor testing are summarized in **Table S2**.

Table S1. Composition by ICP-OES, BET surface areas (S_{BET}) by N_2 physisorption, and copper dispersion (D_{Cu}) by N_2O titration of the $CuGaZrO_x$ samples.

Sample[1]	Cu (wt%)	Ga (wt%)	Zr (wt%)	S_{BET} (m^2 g^{-1})	D_{Cu} (%)
$Cu\text{-}ZrO_x$	24	0	47	112	1.0
$10Cu\text{-}ZrO_x$[2]	9	0	57	109	1.7
$Cu\text{-}GaZrO_x$-48	19	9	36	166	1.0
$Cu\text{-}GaZrO_x$-24	23	22	20	217	1.6
$Cu\text{-}GaO_x$	22	45	0	153	3.1
$GaZrO_x$	0	48	25	131	N/A

[1] The Z in $Cu\text{-}GaZrO_x$-Z refers to the percentage of the molar ratio Zr/(Zr + Cu + Ga).

[2] This sample contains substantially less Cu than the other Cu-containing samples and was only examined due to the poor DRIFTS signal on $Cu\text{-}ZrO_x$.[2] It was synthesized using the same procedure,[1] but using a precursor ratio of $(Cu(NO_3)_2{\cdot}3H_2O)/(ZrO(NO_3)_2{\cdot}H_2O)$ = (0.45 g)/(3.2 g).

Table S2. Methanol space-time yield (*STY*) at iso weight hourly space velocity (*WHSV*) of 48,000 mL(STP) g_{cat}^{-1} h^{-1} and selectivity at isoconversion over the $CuGaZrO_x$ samples.[1] Reactor pressure: 35 bar. Inlet H_2/CO_2: 4/1.

Sample	Activity at iso *WHSV*	Selectivity at isoconversion	
	Methanol *STY* (g_{MeOH} g_{cat}^{-1} h^{-1})	CO_2 conversion (%)	Methanol selectivity (%)
$Cu\text{-}ZrO_x$	0.31 ± 0.02	6.1 ± 0.1	50.9 ± 0.8
$Cu\text{-}GaZrO_x$-48	0.51 ± 0.01	6.6 ± 0.1	58.7 ± 1.0
$Cu\text{-}GaZrO_x$-24	0.63 ± 0.02	7.7 ± 0.6	59.5 ± 1.1
$Cu\text{-}GaO_x$	0.48 ± 0.02	6.8 ± 0.3	53.5 ± 3.1
$GaZrO_x$	0.02 ± 0.01	0.4 ± 0.1 (too low activity)	35.2 ± 0.1

1.2. Experimental setup

The ME-DRIFTS-MS experiments were conducted using a homebuilt setup as shown in **Figure S1**. The setup was capable of introducing concentration perturbations in the DRIFTS cell (Pike DiffusIR with the high-pressure adaptation, modified to reduce the dead volume as described in the estimation of the dead volume and time section below) by switching the positions of 4-way valves (VICI ED6UW). There were three 4-way valves to introduce perturbations in CO_2, H_2, and D_2 concentrations. This design allowed for an active component to be switched off without affecting the total flowrate going to DRIFTS cell since the active component could be replaced by Ar at the same flowrate. Flowrates were set by calibrated mass flow controllers (MFCs, Bronkhorst EL-FLOW Prestige FG-201CV). The pressure of the DRIFTS cell and waste lines were set using mechanical backpressure regulators (BPR, Equilibar ZF1SNN8). All four BPRs were capable of setting the working pressure from 1 to 50 bar (absolute) using a single reference line. This reference line was controlled by an electronic back pressure regulator pilot (Equilibar EPR-1000). The outlet from the main BPR went to a mass spectrometer (MS, Extrel MAX300-CAT). All the lines and

the BPR from the DRIFTS cell to the MS were kept at 140 °C to avoid condensation of products. A pressure gauge (Bronkhorst EL-PRESS P-502C) was located upstream of the DRIFTS cell to measure the pressure drop across the DRIFTS cell and was utilized to identify leakage and cloggage.

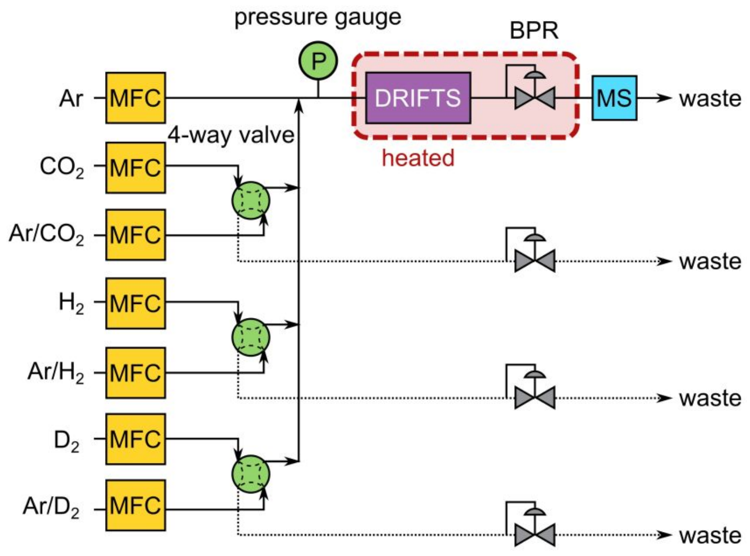

Figure S1. Simplified schematic of the ME-DRIFTS-MS setup. MFC: mass flow controller. BPR: back pressure regulator.

1.3. ME-DRIFTS-MS experiments

10 mg of sample powder was loaded on top of a ceramic sample cup partially filled with KBr powder (FT-IR grade). This configuration ensured that the sample cups were filled to the same height using the same mass of samples despite their differences in density. All reported temperatures refer to the temperature at the sample surface, empirically corrected to account for the temperature gradient in the sample cup. Samples were reduced *in-situ* at 1 bar and 260 °C (5 °C min^{-1} ramp rate) in 20% H_2/Ar for approximately 2 hours prior to the DRIFTS experiments. Then, the working pressure and flowrates were set according to **Table S3** at 260 °C unless otherwise noted. A background spectrum was collected at this point, and the MES experiment was carried out. The MES experiments typically consisted of 6 cycles and modulated the flowrate of CO_2 while keeping that of H_2 constant. Within one cycle, the IR spectrometer (Bruker Vertex 70 equipped with a mercury cadmium telluride (MCT) detector) collected in the Rapid Scan mode 60 spectra while CO_2 was flowing to the DRIFTS cell and 60 additional spectra while CO_2 was being vented. All spectra were collected with a resolution of 4 cm^{-1}. The modulations were fully automated using a voltage-based communication protocol between the spectrometer trigger box (Bruker S 129/Z) and the 4-way switching valves. The time period was varied by setting different scans per spectra according to **Table S3**. The MS collected simultaneously at the outlet all *m/z* signals between 1 and 60. MS signals were normalized by that of (*m/z* 2) since the inlet F_{H2} was constant during the MES experiments.

Modulating CO_2 flowrate while keeping that of H_2 constant enables the reaction and catalyst regeneration to alternate in each half cycle.[3–5] If the H_2 feed was alternatively switched on and off while keeping the CO_2 feed constant, stable species (e.g., carbonates and formates) might build up and inhibit active sites.[6,7] We verified this hypothesis experimentally by modulating H_2 instead of CO_2 over Cu-GaZrO$_x$-24 at 20 bar and 260 °C (**Figure S14**), and the resulting DRIFTS spectra showed a decrease in the relative CH_3OH(g)/CO(g) IR signal from 11.25 to 0.38 as well as a disappearance of an IR signal associated with Ga–H.

Table S3. Implemented flowrates, period times, and averaged scans per spectra at the different working pressures.

P **(bar)**	F_{H2} **(sccm)**	F_{CO2} **(sccm)**	F_{Ar} **(sccm)**	***T*** **(min)**	**Scans per spectra**	**Time per spectra (s)**
1	16	4	0	6	32	3
20	30	7.5	25	12	64	6
35	40	10	25	24	128	12
50	44	11	25	48	256	24

1.4. MES-PSD analysis of DRIFTS data

A baseline correction algorithm was applied to the time-domain DRIFTS spectra due to thermal fluctuations (arising from heats of reaction and differences in heat capacity between the inlet gas of the two half cycles), assuming the region between 2600 and 2700 cm^{-1} contained no signals. A deglitching algorithm was also applied to remove any data points that were 3 standard deviations away from the mean as outliers. The DRIFTS spectra of the first two cycles were discarded to ensure perturbations were around a quasi-steady state. The remaining spectra were averaged as one cycle and then transformed from the time domain to the phase domain according to (**eq. 4**) of the manuscript, utilizing a fast Fourier transform (FFT) algorithm.[8]

1.5. Methanol steam reforming experiments

10 mg of sample powder was loaded onto a sample cup as described above. The samples were reduced *in-situ* at 1 bar and 260 °C (5 °C min^{-1} ramp rate) in 20% H_2/Ar for approximately 2 hours. The inlet gas was switched to 20 sccm Ar for at least 30 minutes to purge weakly bound H_2 and the lines. A background spectrum was collected at this point. DRIFTS spectra were collected repeatedly every minute at 128 scans per spectra and 4 cm^{-1} spectral resolution. For the first 30 minutes, the 20 sccm Ar was directed into a bubbler filled with methanol at 10 °C prior to its entry to the DRIFTS cell at 260 °C (**Figure S2**). At the 30-minute mark, the bubbler was bypassed for at least 30 minutes to monitor the stability of the adsorbed species.

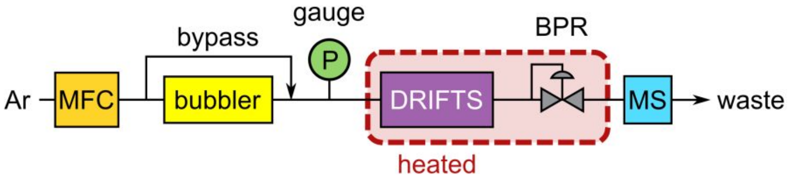

Figure S2. Simplified schematic for the setup used for the methanol steam reforming experiments. MFC: mass flow controller. BPR: back pressure regulator.

1.6. Reference DRIFTS spectra

Reference DRIFTS spectra of (bi)carbonates and formates were collected on precipitated Ga_2O_3 and ZrO_2 by flowing CO_2 and CO, respectively. 10 mg of sample powder was loaded onto a sample cup as described above. The samples were dehydrated *in-situ* in pure Ar at 1 bar and 400 °C (5 °C min^{-1} ramp rate). Then, the samples were cooled down to 260 °C, and the working pressure was raised to 20 bar. A background spectrum was collected at this point. DRIFTS spectra were collected repeatedly every minute at 128 scans per spectra and 4 cm^{-1} spectral resolution. The inlet gas was set to 7.5 sccm CO_2 (or CO) and 55 sccm Ar for 15 minutes. Then, the inlet gas was switched to 7.5 sccm CO_2 (or CO), 30 sccm H_2, and 25 sccm Ar for an additional 15 minutes. Finally, the inlet gas was switched to pure Ar for at least 30 minutes to monitor the stability of the adsorbed species.

1.7 Temperature-programmed desorption of water

Temperature-programmed desorption of water was performed using a coupled thermogravimetric analysis–differential scanning calorimetry–mass spectrometry (TGA–DSC–MS) system. TGA–DSC measurements were performed on a Mettler Toledo TGA/DSC 1 instrument, with its effluent continuously directed into a Pfeiffer Omnistar GSD 320 mass spectrometer. Approximately 40 mg of $CuGaZrO_x$-24 was reduced under a 60 sccm flow of 4% H_2/N_2 and heated to 260 °C at a ramp rate of 5 °C min^{-1}. The temperature was then held at 260 °C for 2 hours under the reducing conditions before switching the flowing gas to 60 sccm N_2 while maintaining the same temperature for 30 minutes. Finally, the temperature was increased to 800 °C at a ramp rate of 20 °C min^{-1} under a constant N_2 flow. Water desorption was monitored by measuring (m/z 18) signal in the mass spectrometer.

2. Reference DRIFTS spectra on single components

2.1. (Bi)carbonates on ZrO_2 and Ga_2O_3

The reference spectra of carbonates and bicarbonate were collected by flowing CO_2 over Ga_2O_3 and ZrO_2 (**Figure S3**). The asymmetric vibrations were the most intense signals, centered between 1621 and 1700 cm^{-1}. The symmetric vibrations were found to be centered around 1267 cm^{-1} on both oxides.[9] ZrO_2 additionally exhibited broad peaks in the OCO region. After the introduction of H_2 in the feed, some broadening of the peaks and evolution of minor peaks in the C–H stretching region could be observed, especially on Ga_2O_3.

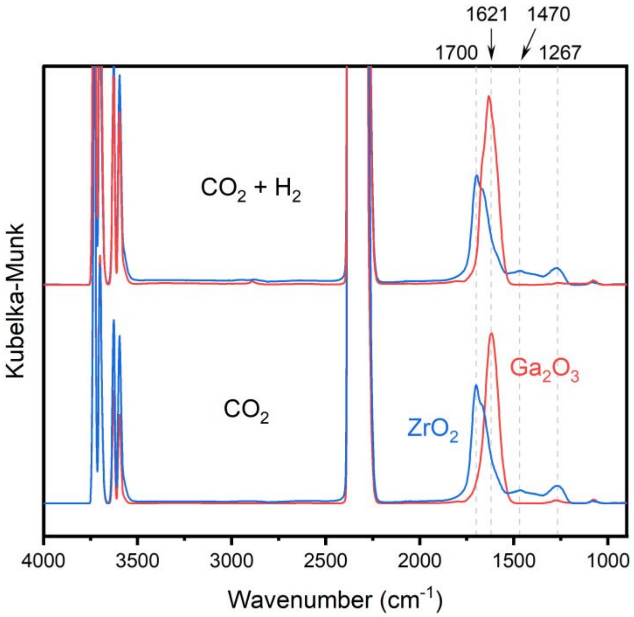

Figure S3. Reference spectra of carbonates and bicarbonates on precipitated Ga_2O_3 and ZrO_2 at 260 °C and 20 bar.

2.2. Formates on ZrO_2 and Ga_2O_3

The reference spectra of formates were collected by flowing CO over Ga_2O_3 and ZrO_2. Formates could be generated by the reaction between surface hydroxyls and CO,[10] as shown below:

OH + CO → HCOO

Figure S4a shows C–H stretching peaks on ZrO_2 centered at around 2859 cm^{-1}, especially after the introduction of H_2, which indicates the successful generation of formates from CO on this oxide. Asymmetric OCO vibrations between 1582 and 1641 cm^{-1} and symmetric ones centered at around 1290 cm^{-1} and 1381 cm^{-1} were also observable. For Ga_2O_3, the asymmetric OCO vibrations were more easily observable. The accompanying C–H stretching could be observed as a minor peak centered at around 2887 cm^{-1} (**Figure S4b**).

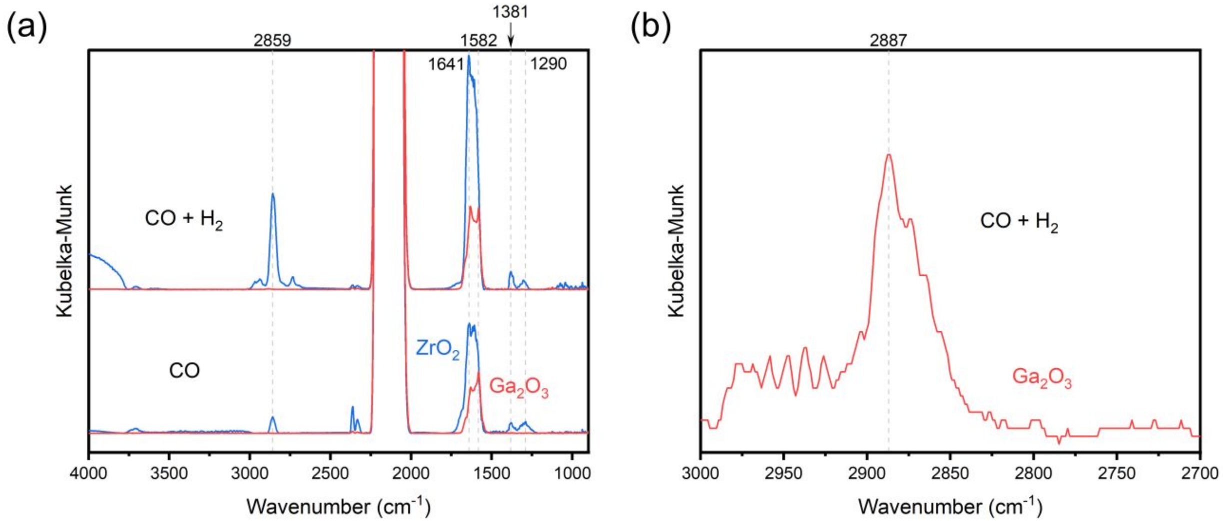

Figure S4. (a) Reference DRIFTS spectra of formates on precipitated Ga_2O_3 and ZrO_2 at 260 °C and 20 bar. (b) Zoomed-in DRIFTS spectra to show the C–H stretching on Ga_2O_3.

2.3. Methoxy on ZrO_2 and Ga_2O_3

Reference spectra of methoxy (CH_3O^*) were acquired by directly flowing methanol on Ga_2O_3 and ZrO_2 (**Figure S5**). The C-O stretching of CH_3O^* was found to be centered at 1071 cm^{-1} on Ga_2O_3, as evidenced by the spectra collected after switching off the bubbler (to remove $CH_3OH(g)$ signals). While this peak was still observed on ZrO_2, the main C–O stretching peaks were at 1111 and 1154 cm^{-1}.[11] These were accompanied by multiple C–H stretching peaks centered between 2797 and 2935 cm^{-1}.

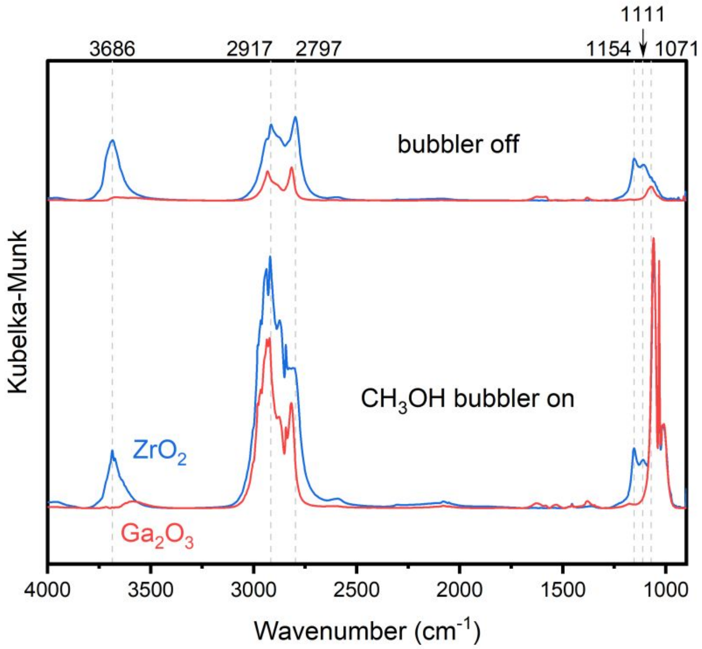

Figure S5. Reference spectra of methoxy on precipitated Ga_2O_3 and ZrO_2 at 260 °C and 1 bar.

3. Estimation of dead volume and time

To estimate the dead volume and time of the ME-DRIFTS-MS setup, we introduced a step change in the feed from 20 sccm Ar to 20 sccm CO_2 by switching the corresponding 4-way valve (**Figure S1**). This Ar-to-CO_2 step change is the basis of our MES experiments (since H_2 flowrate was kept constant throughout the MES experiments). **Figure S6a** shows that the response at 1 bar in the bypass mode (*i.e.*, bypassing the DRIFTS cell in **Figure S1**) resembles that of plug flow with just 26 seconds of dead time. With the Pike DRIFTS cell attached, the response deviates from ideality. We therefore defined the variable $t_{50\%}$ as the time it takes for the MS to measure at least 50% of the normalized CO_2 signal $(I_{m/z\ 44})/(I_{m/z\ 44} + I_{m/z\ 40})$ at the outlet. This value was 64 seconds with the DRFITS cell attached. We then filled some of the dead volume of the DRIFTS cell with machinable aluminum silicate (Lava) (inset of **Figure S6a**). This reduced the dead time $t_{50\%}$ of the Ar-to-CO_2 step function marginally from 64 s to 58 s, highlighting that (back) diffusion is minimal at 1 bar. The response at 35 bar is shown in **Figure S6b**. The $t_{50\%}$ value was 14 minutes in the bypass mode. When the unmodified DRIFTS cell was attached, $t_{50\%}$ became 50 minutes. The Lava pieces in the DRIFTS cell were able to reduce $t_{50\%}$ to 35 minutes. This observation highlights that the effect of (back) diffusion is more pronounced at high pressures and low flow conditions. Accordingly, our MES experiments at high pressures utilized flowrates that were higher than 20 sccm, to lower dead times and the duration of experiments. We assumed dead times and flowrates were inversely proportional. We targeted half-period times ($T/2$) that were at least equal to the value of $t_{50\%}$ at the corresponding pressure and flowrate (**Table S4**). This allowed enough time for the gases to be exchanged in the DRIFTS cell and the lines between the half cycles.

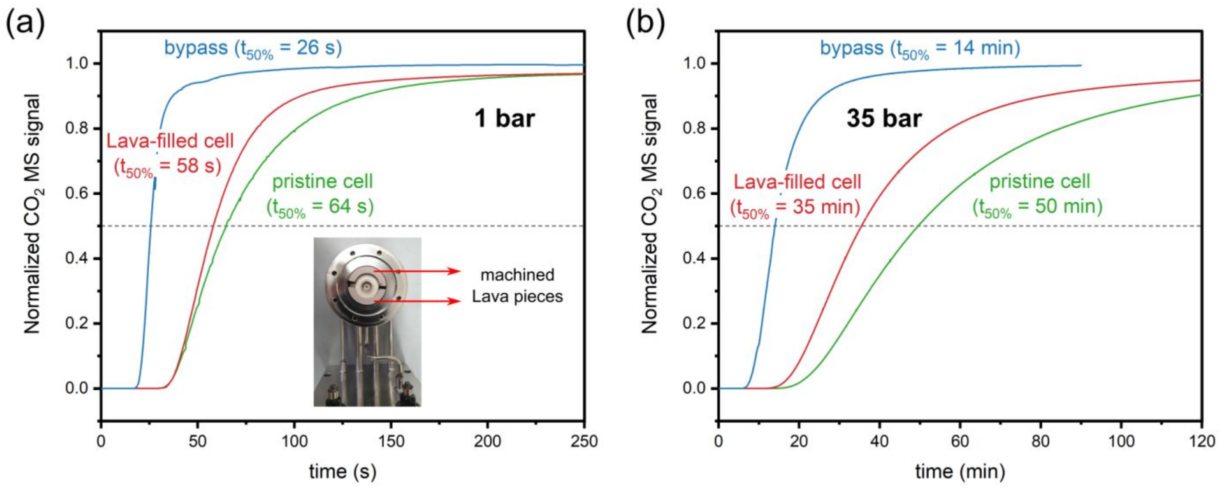

Figure S6. Normalized CO_2 MS signal of a step change from 20 sccm Ar to 20 sccm CO_2 at (a) 1 bar and (b) 35 bar. The inset of (a) shows the PIKE cell filled with Lava pieces.

Table S4. Prediction of dead times ($t_{50\%}$) at the different pressures and flowrates of the MES experiments. Predicted $t_{50\%}$ values are based on the setup with the Lava-filled DRIFTS cell.

P_{total} (bar)	F_{total} (sccm)	Predicted $t_{50\%}$ (min)	*Set T/2* (min)
1	20	1	3
20	62.5	6	6
35	75	9	12
50	80	13	24

4. Phase-resolved DRIFTS spectra of CO_2 hydrogenation MES experiments on $CuGaZrO_x$

4.1. $Cu-GaO_x$

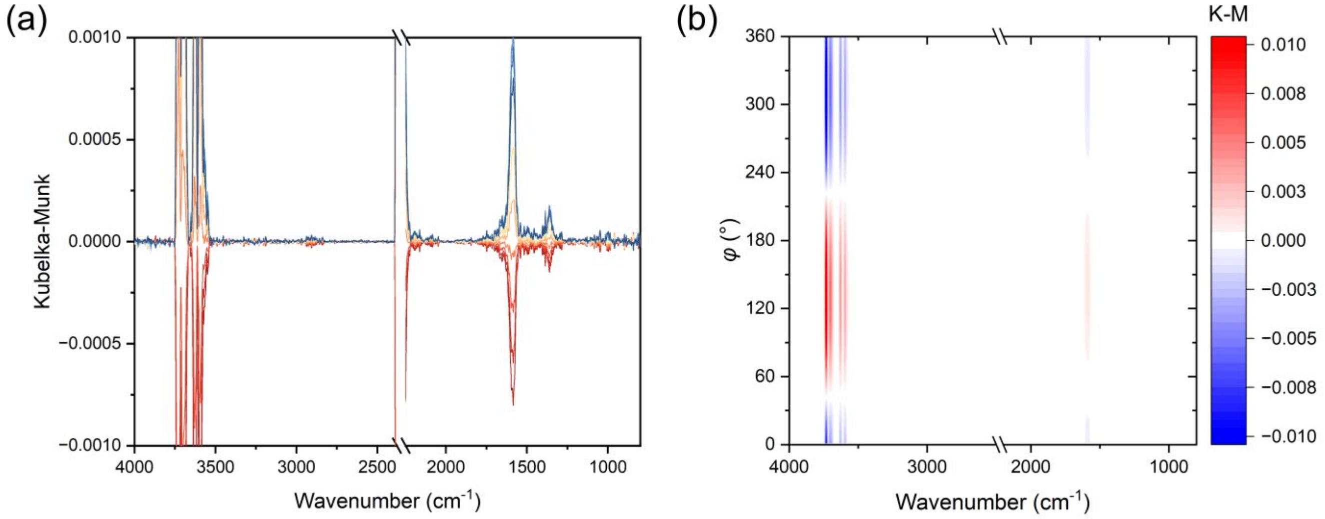

Figure S7. Phase-resolved DRIFTS spectra of CO_2 hydrogenation on $Cu-GaO_x$ at 1 bar and 260 °C.

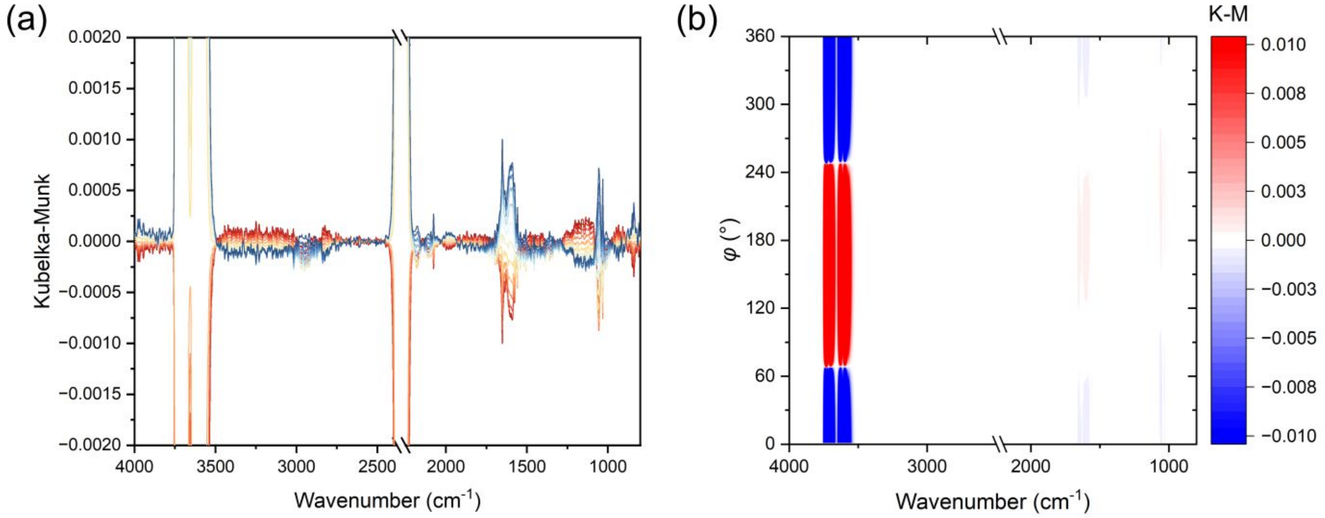

Figure S8. Phase-resolved DRIFTS spectra of CO_2 hydrogenation on $Cu-GaO_x$ at 20 bar and 260 °C.

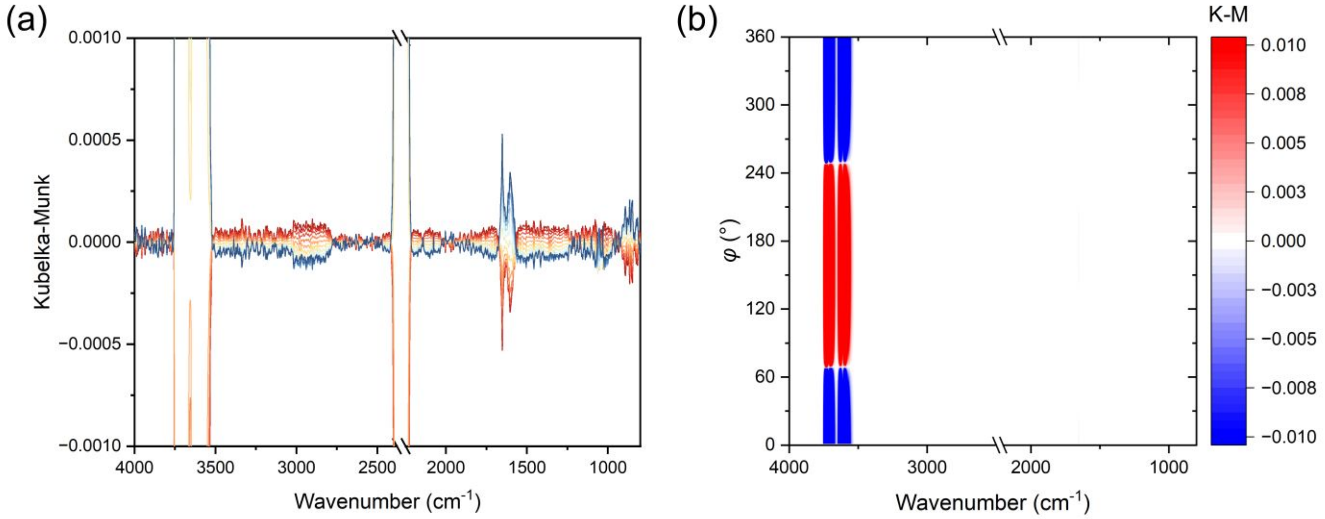

Figure S9. Phase-resolved DRIFTS spectra of CO_2 hydrogenation on Cu-GaO$_x$ at 20 bar and 240 °C.

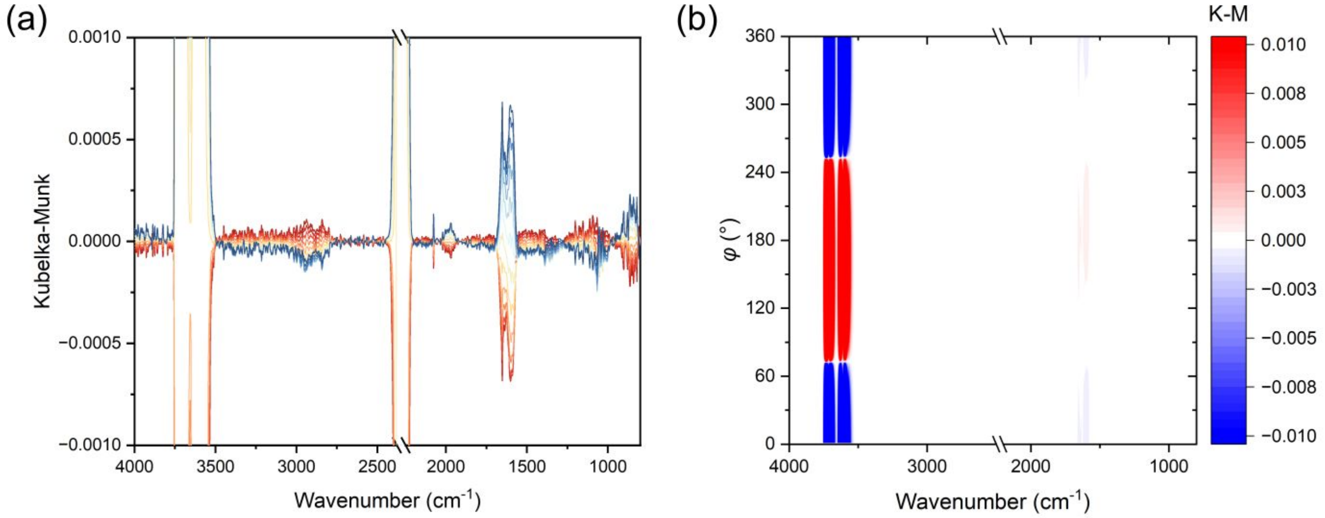

Figure S10. Phase-resolved DRIFTS spectra of CO_2 hydrogenation on Cu-GaO$_x$ at 20 bar and 220 °C.

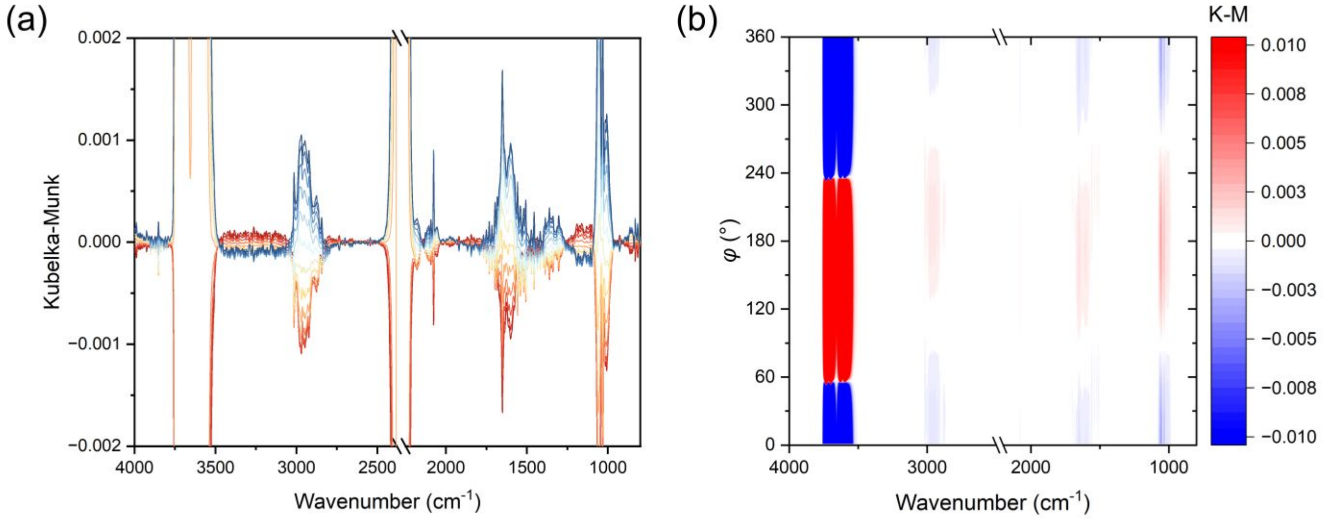

Figure S11. Phase-resolved DRIFTS spectra of CO_2 hydrogenation on Cu-GaO_x at 35 bar and 260 °C.

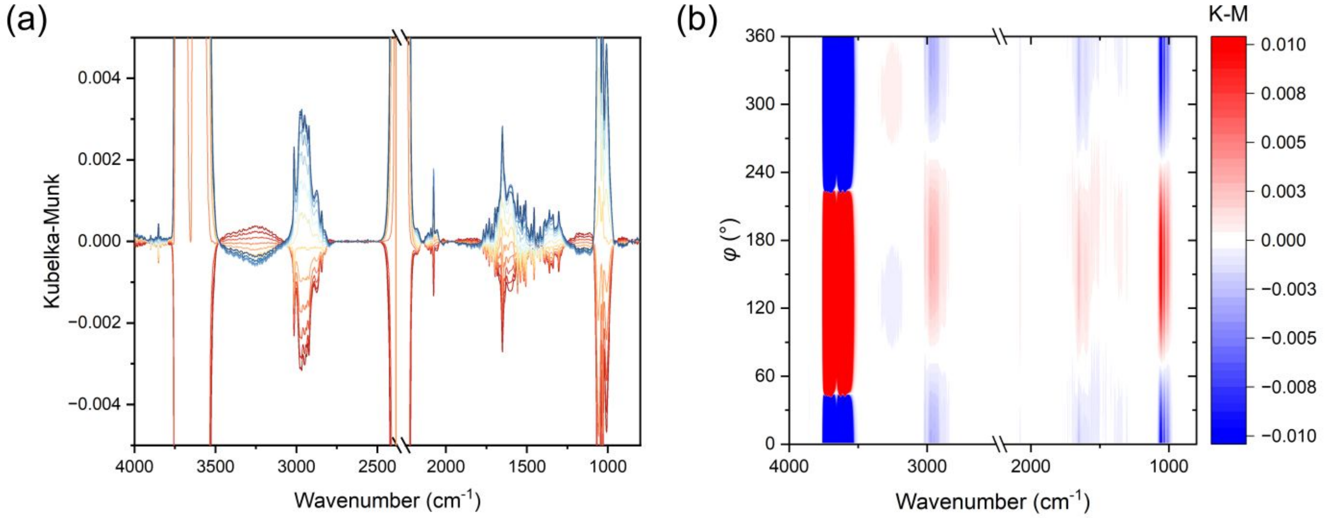

Figure S12. Phase-resolved DRIFTS spectra of CO_2 hydrogenation on Cu-GaO_x at 50 bar and 260 °C.

4.2. Cu-GaZrO$_x$-24

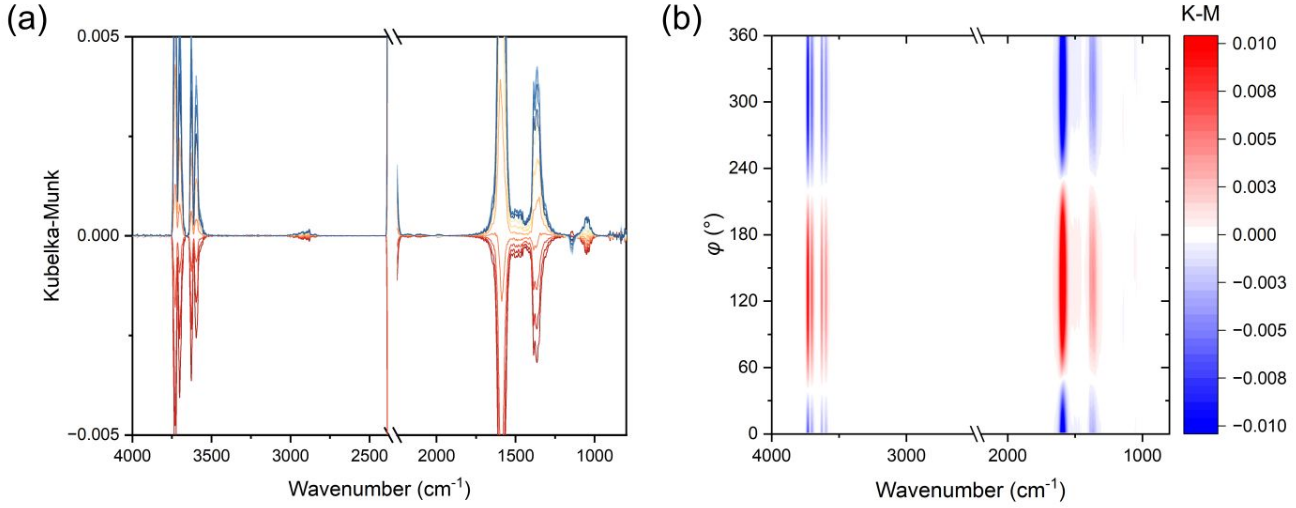

Figure S13. Phase-resolved DRIFTS spectra of CO_2 hydrogenation on Cu-GaZrO$_x$-24 at 1 bar and 260 °C.

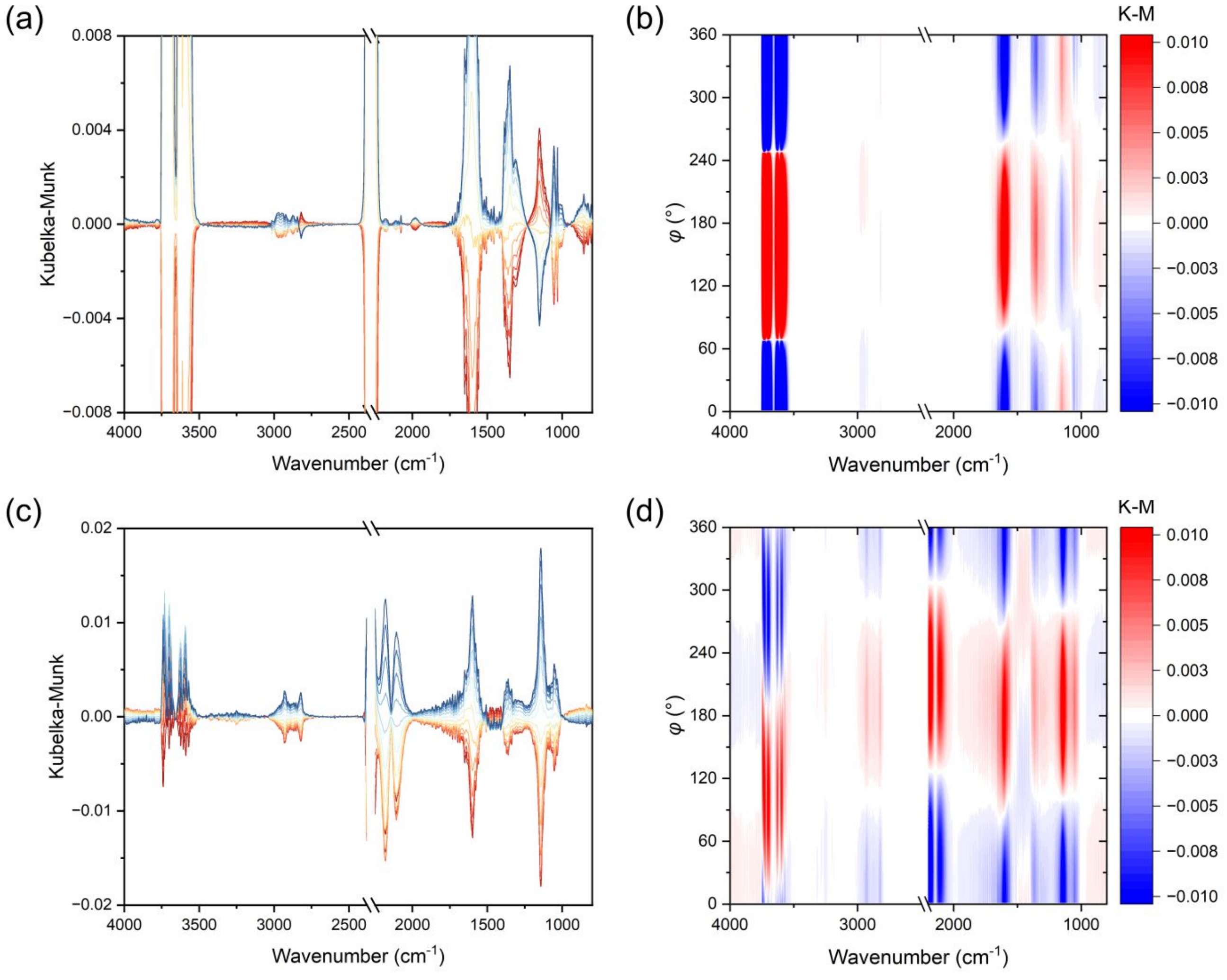

Figure S14. Phase-resolved DRIFTS spectra of CO_2 hydrogenation on Cu-GaZrO$_x$-24 at 20 bar and 260 °C by modulating (a-b) inlet CO_2 and (c-d) inlet H_2.

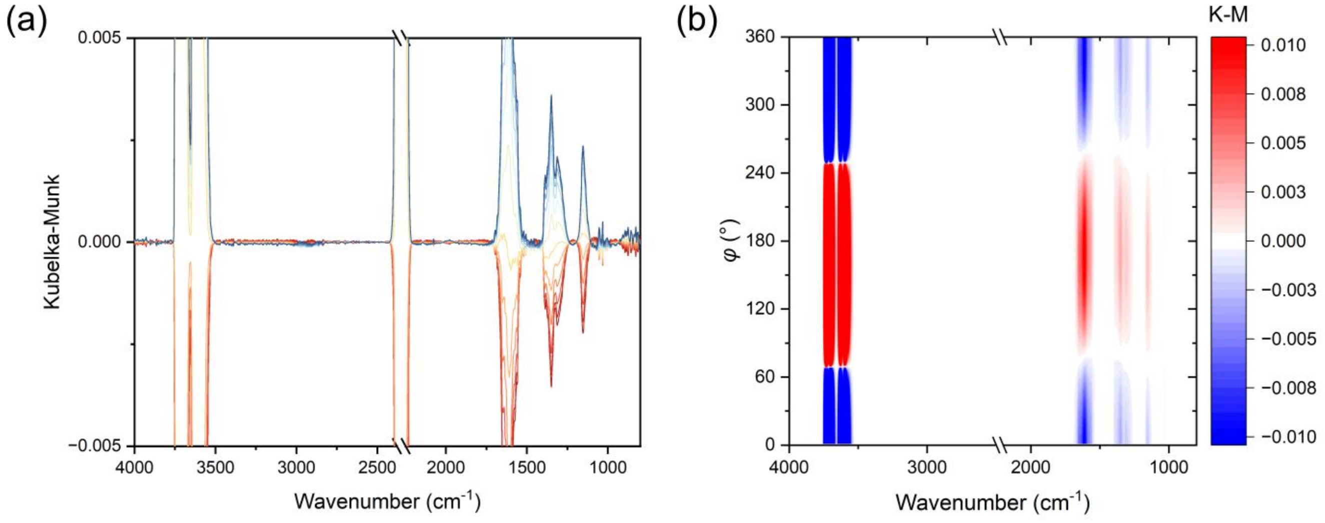

Figure S15. Phase-resolved DRIFTS spectra of CO_2 hydrogenation on Cu-GaZrO$_x$-24 at 20 bar and 240 °C.

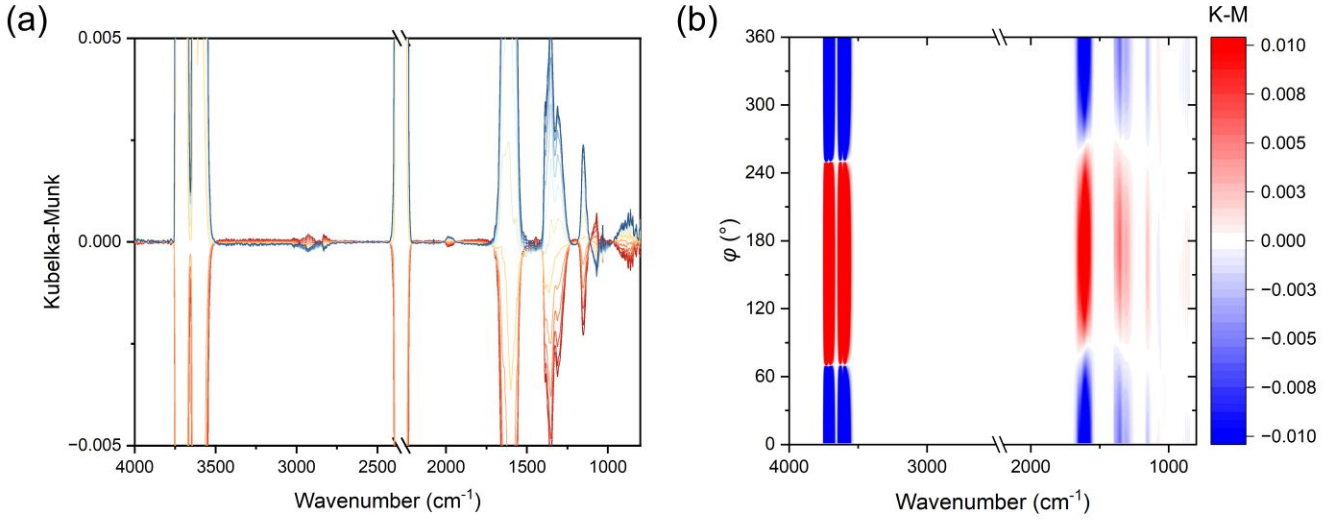

Figure S16. Phase-resolved DRIFTS spectra of CO_2 hydrogenation on Cu-GaZrO$_x$-24 at 20 bar and 220 °C.

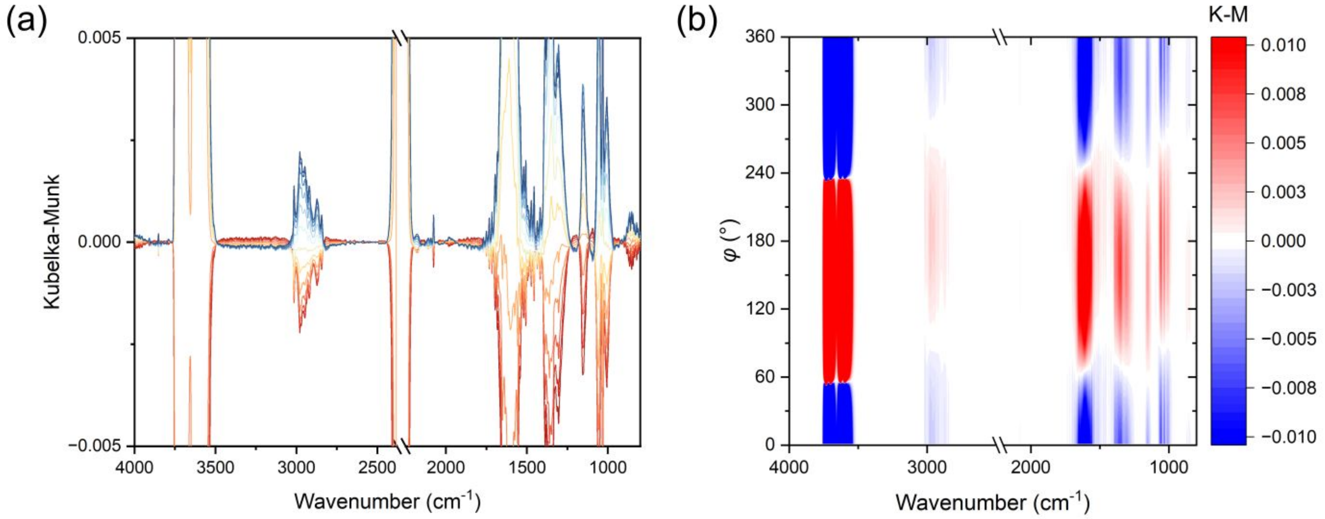

Figure S17. Phase-resolved DRIFTS spectra of CO_2 hydrogenation on Cu-GaZrO$_x$-24 at 35 bar and 260 °C.

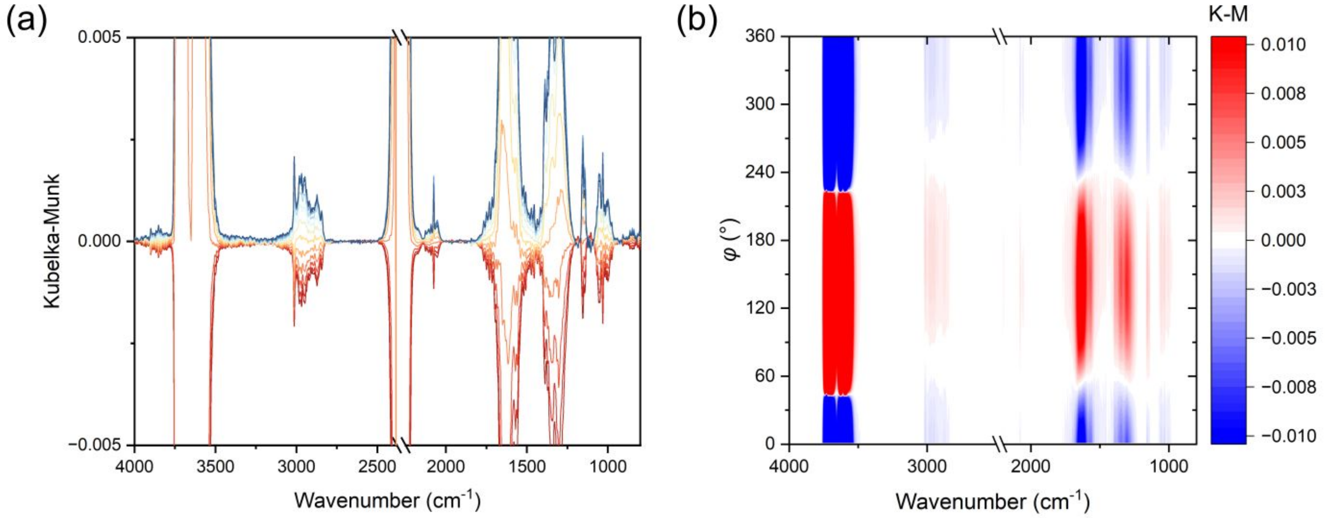

Figure S18. Phase-resolved DRIFTS spectra of CO_2 hydrogenation on Cu-GaZrO$_x$-24 at 50 bar and 260 °C.

4.3. Cu-GaZrO$_x$-48

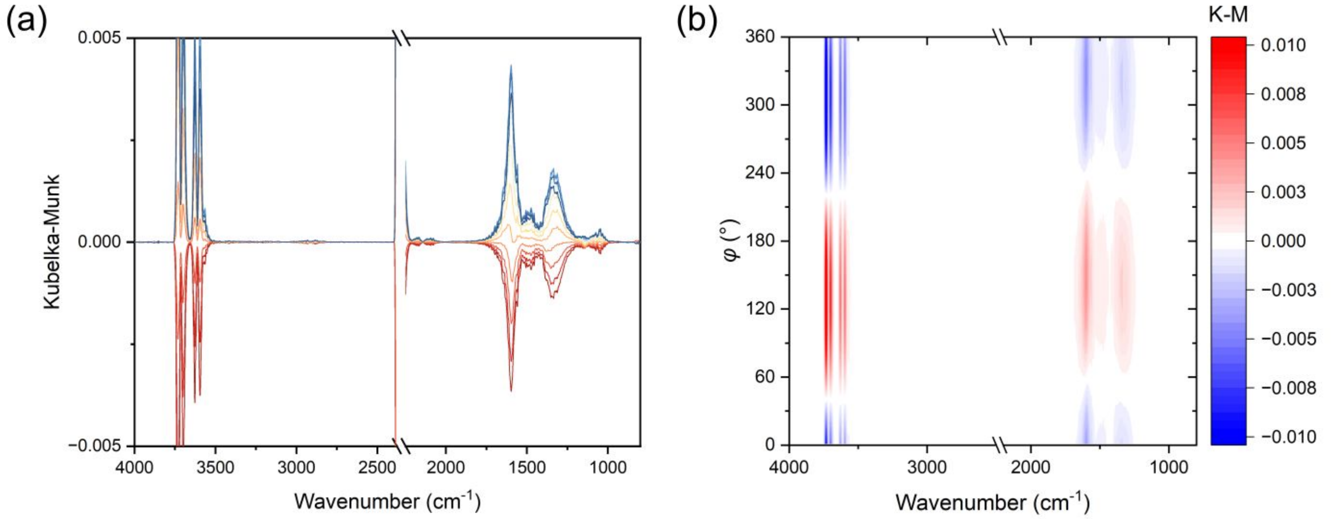

Figure S19. Phase-resolved DRIFTS spectra of CO_2 hydrogenation on Cu-GaZrO$_x$-48 at 1 bar and 260 °C.

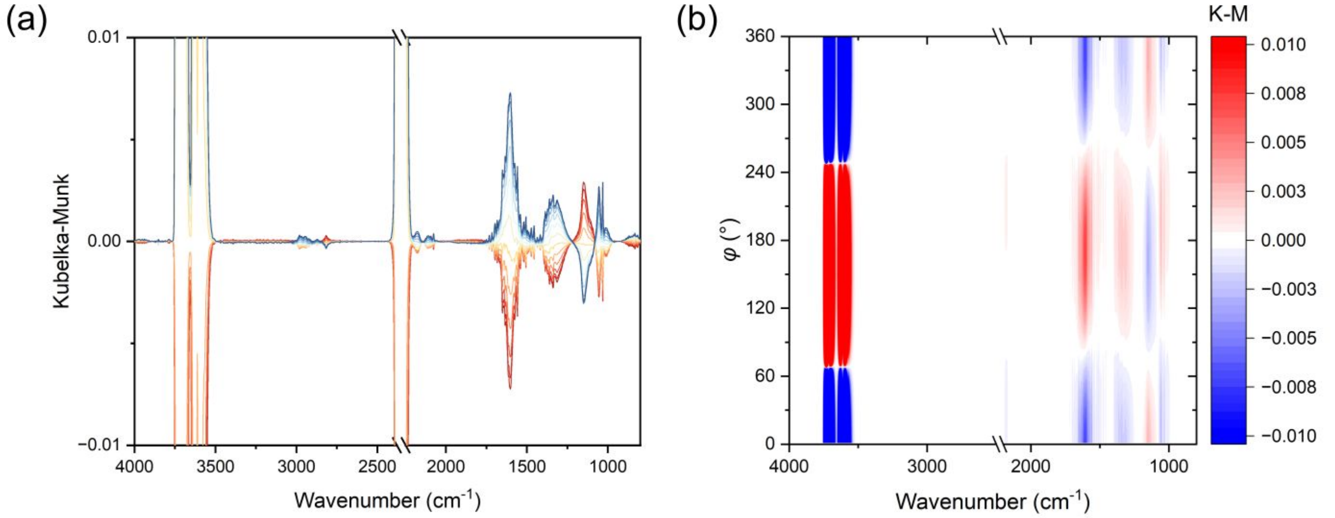

Figure S20. Phase-resolved DRIFTS spectra of CO_2 hydrogenation on Cu-GaZrO$_x$-48 at 20 bar and 260 °C.

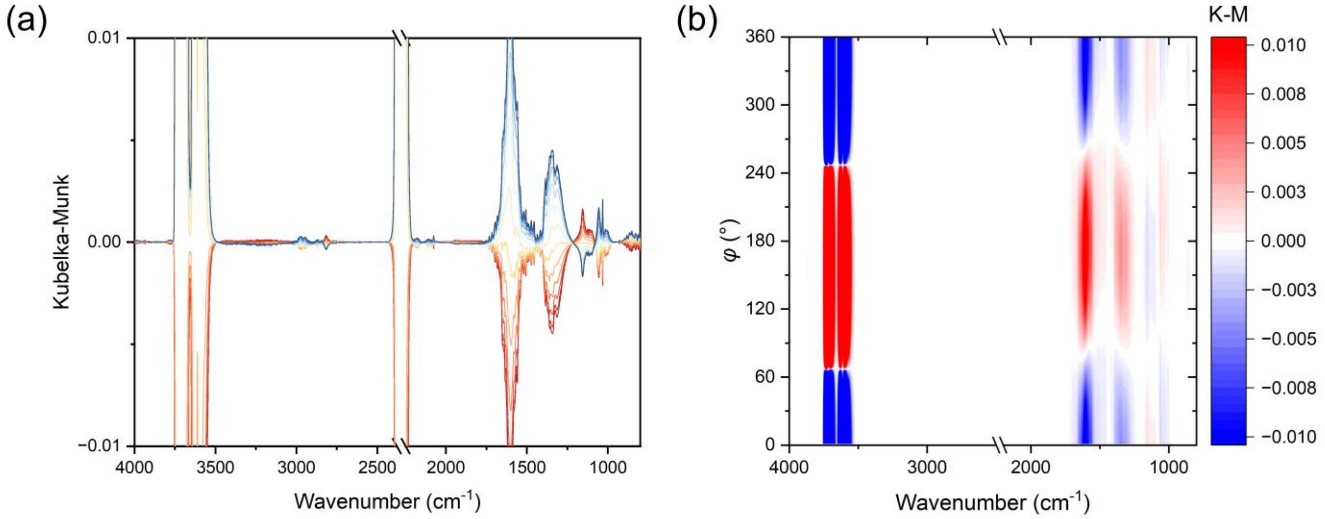

Figure S21. Phase-resolved DRIFTS spectra of CO_2 hydrogenation on Cu-GaZrO$_x$-48 at 20 bar and 240 °C.

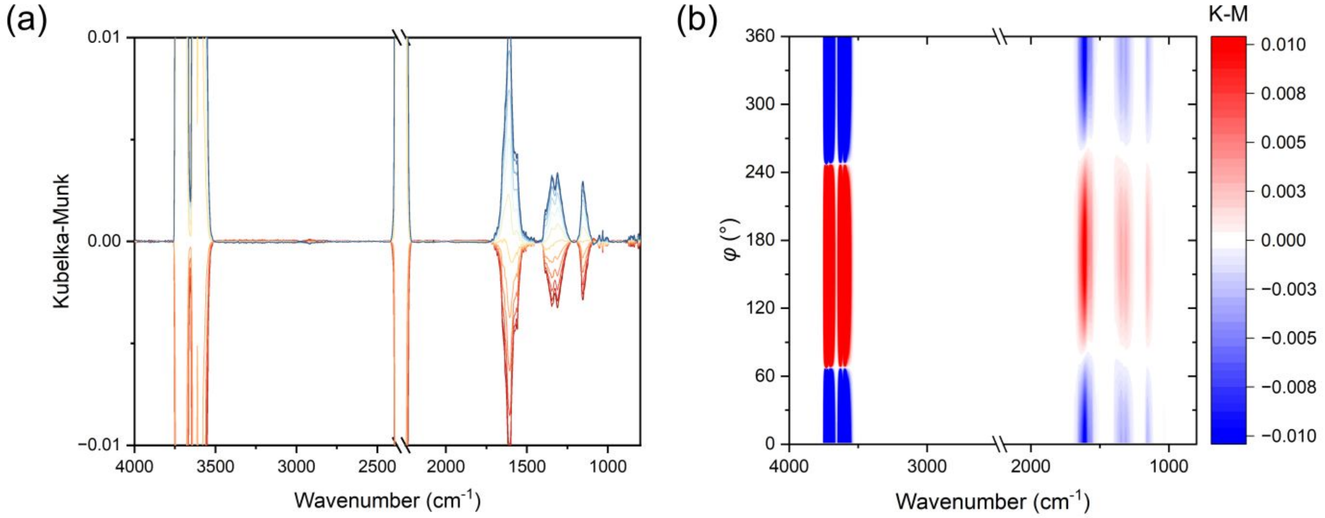

Figure S22. Phase-resolved DRIFTS spectra of CO_2 hydrogenation on Cu-GaZrO$_x$-48 at 20 bar and 220 °C.

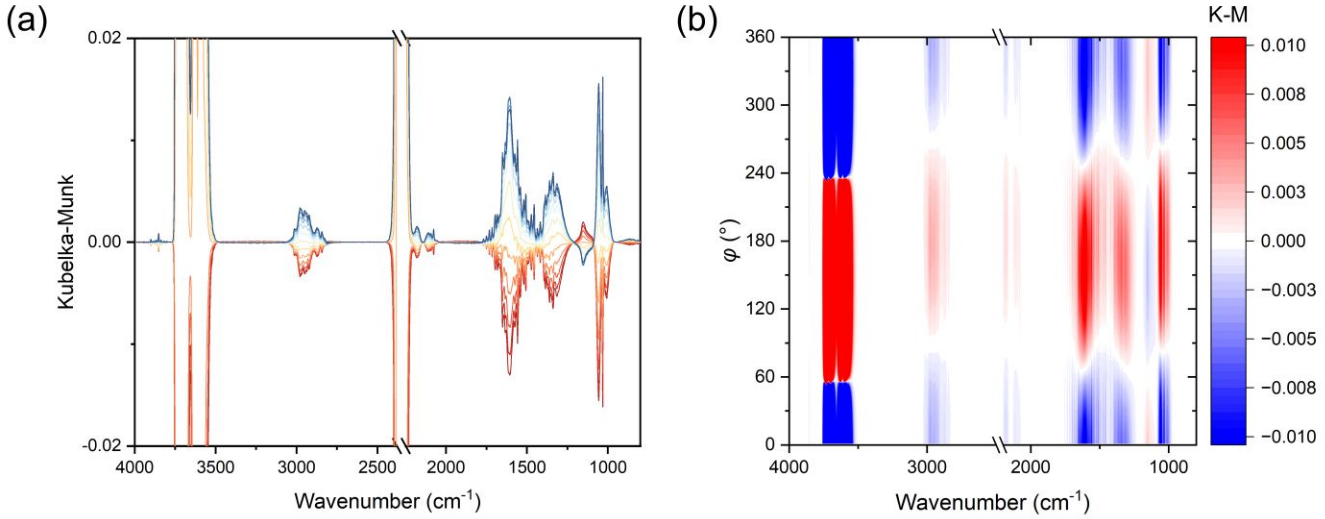

Figure S23. Phase-resolved DRIFTS spectra of CO_2 hydrogenation on Cu-GaZrO$_x$-48 at 35 bar and 260 °C.

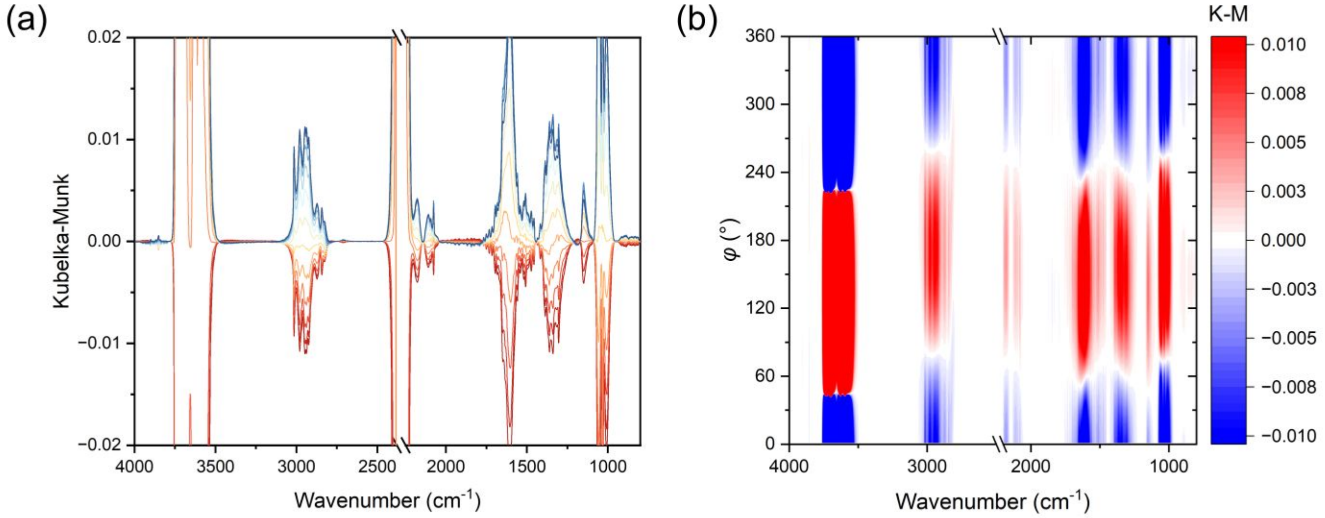

Figure S24. Phase-resolved DRIFTS spectra of CO_2 hydrogenation on Cu-GaZrO$_x$-48 at 50 bar and 260 °C.

4.4. Cu-ZrO_x

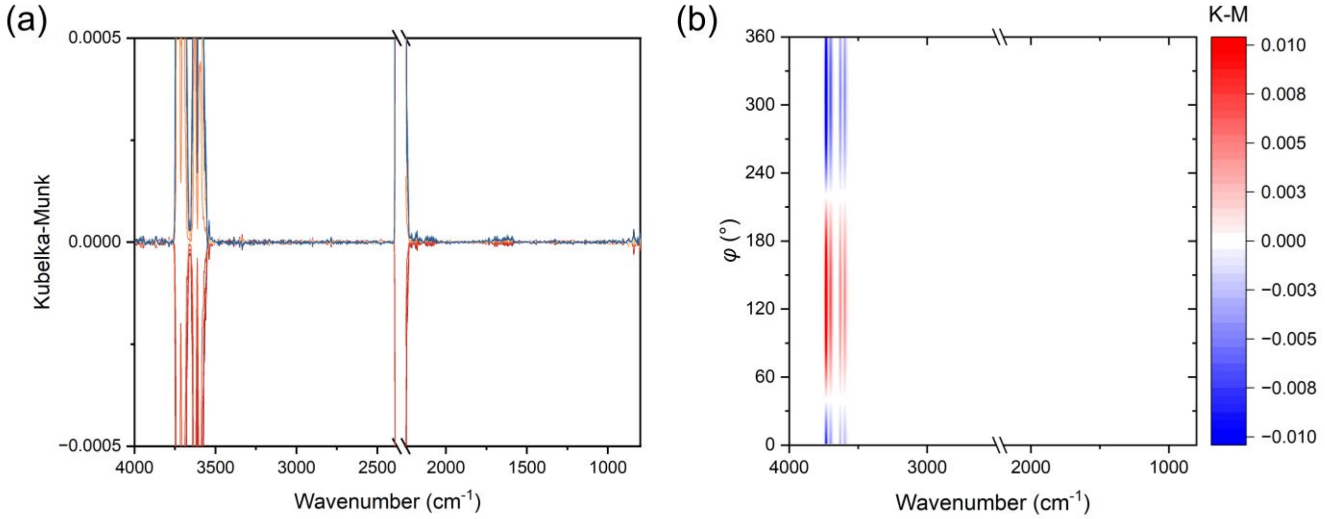

Figure S25. Phase-resolved DRIFTS spectra of CO_2 hydrogenation on Cu-ZrO_x at 1 bar and 260 °C.

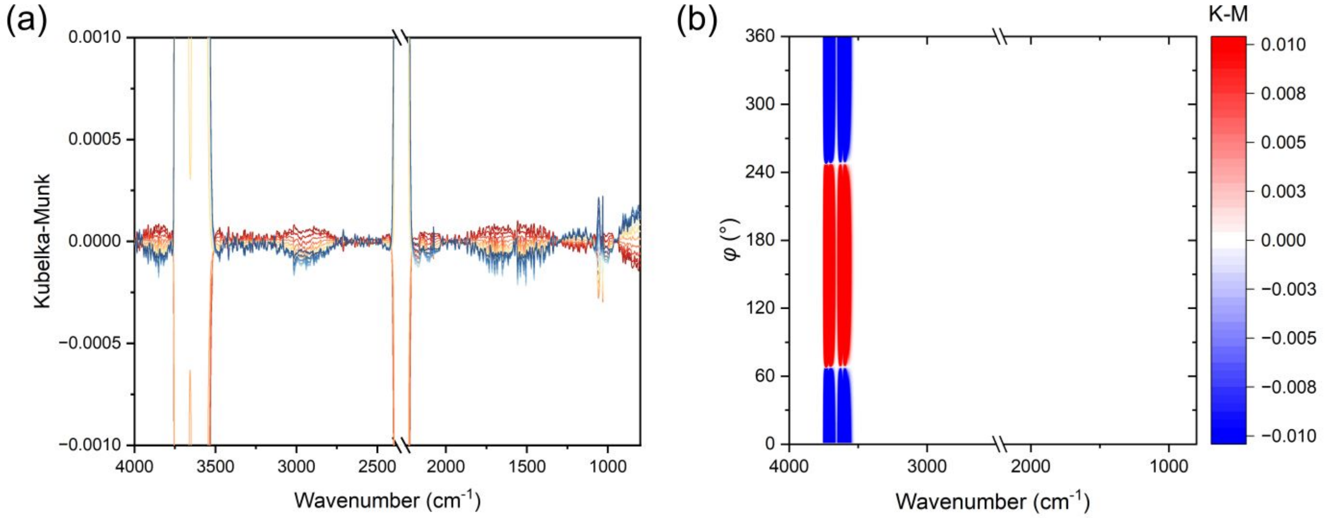

Figure S26. Phase-resolved DRIFTS spectra of CO_2 hydrogenation on Cu-ZrO_x at 20 bar and 260 °C.

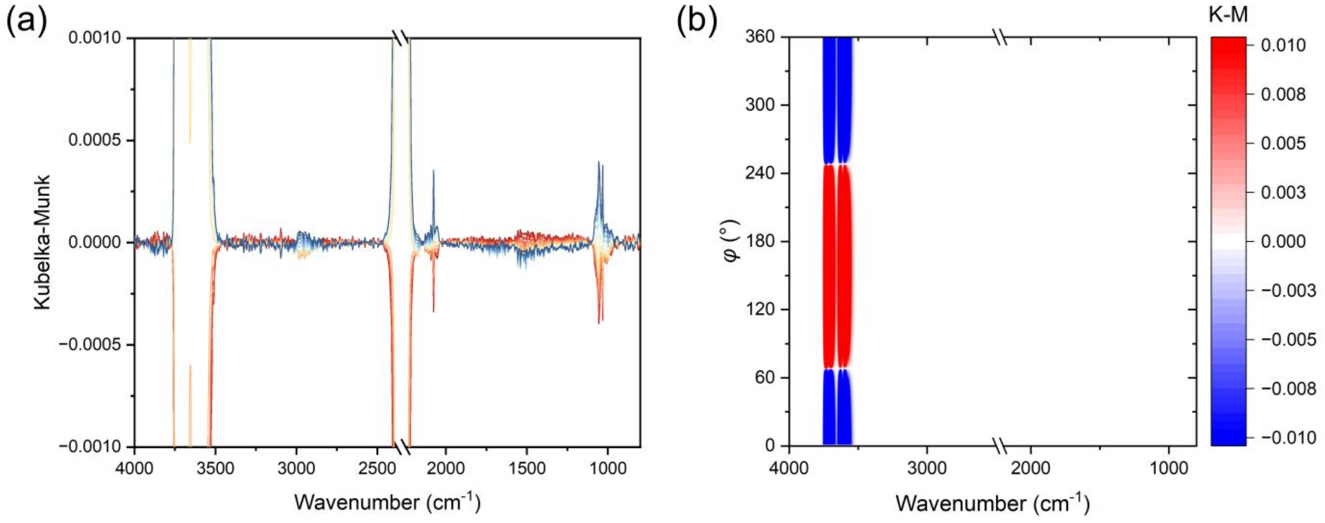

Figure S27. Phase-resolved DRIFTS spectra of CO_2 hydrogenation on Cu-ZrO_x at 20 bar and 240 °C.

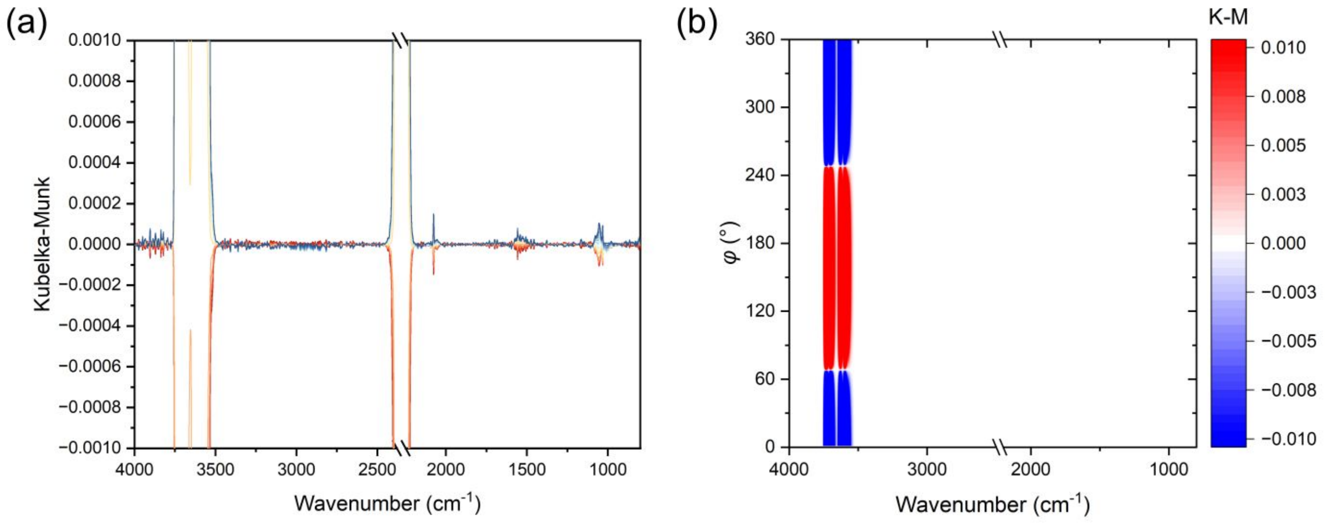

Figure S28. Phase-resolved DRIFTS spectra of CO_2 hydrogenation on Cu-ZrO_x at 20 bar and 220 °C.

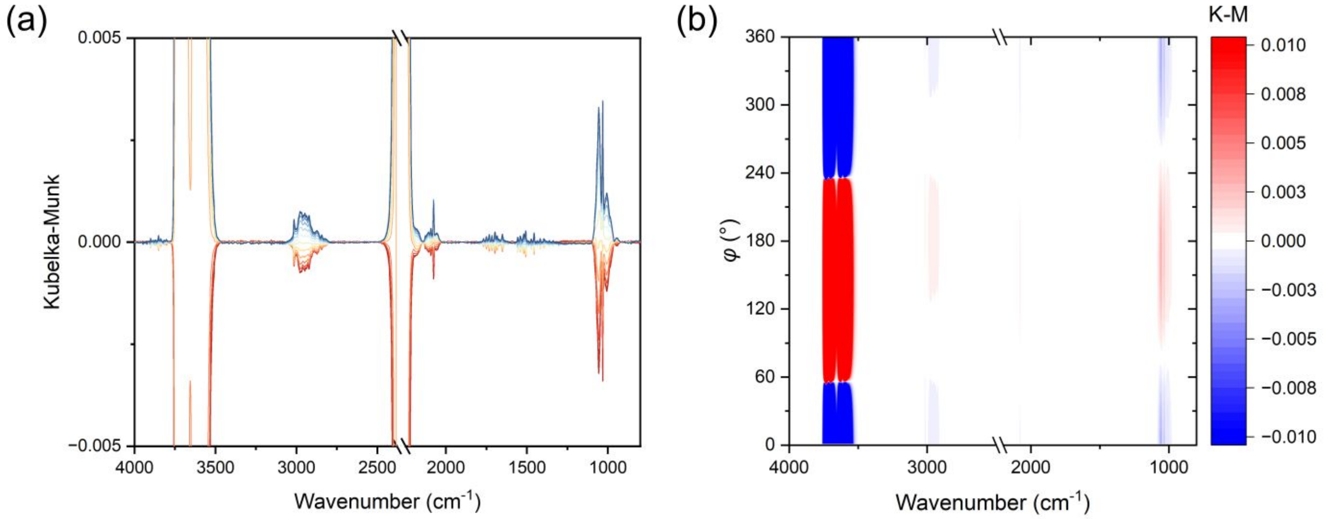

Figure S29. Phase-resolved DRIFTS spectra of CO_2 hydrogenation on Cu-ZrO_x at 35 bar and 260 °C.

4.5. 10Cu-ZrO_x

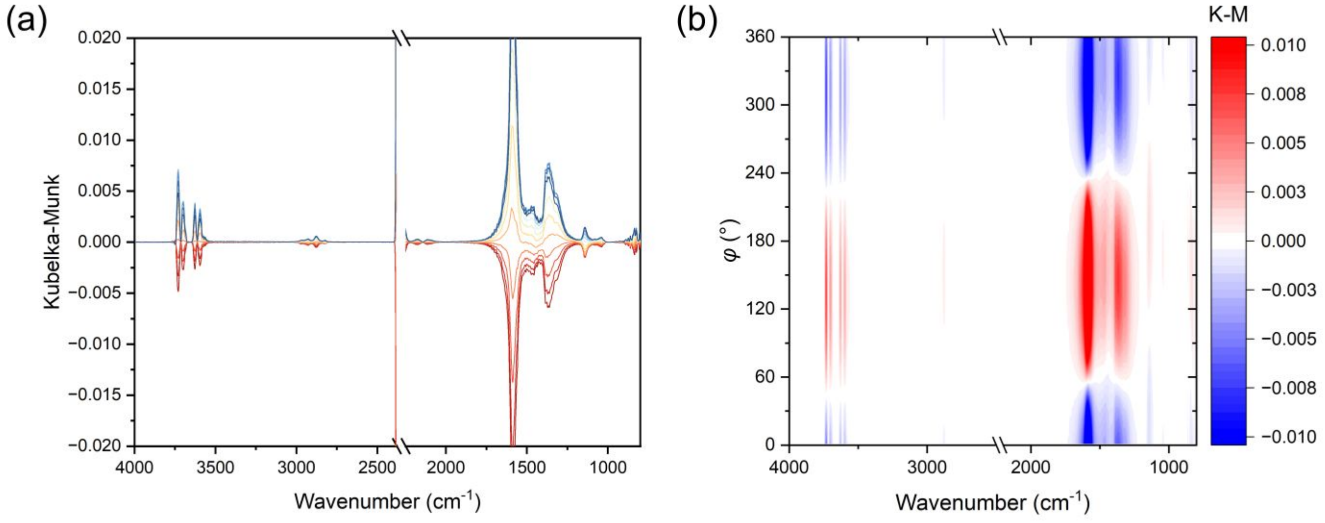

Figure S30. Phase-resolved DRIFTS spectra of CO_2 hydrogenation on 10Cu-ZrO_x at 1 bar and 260 °C.

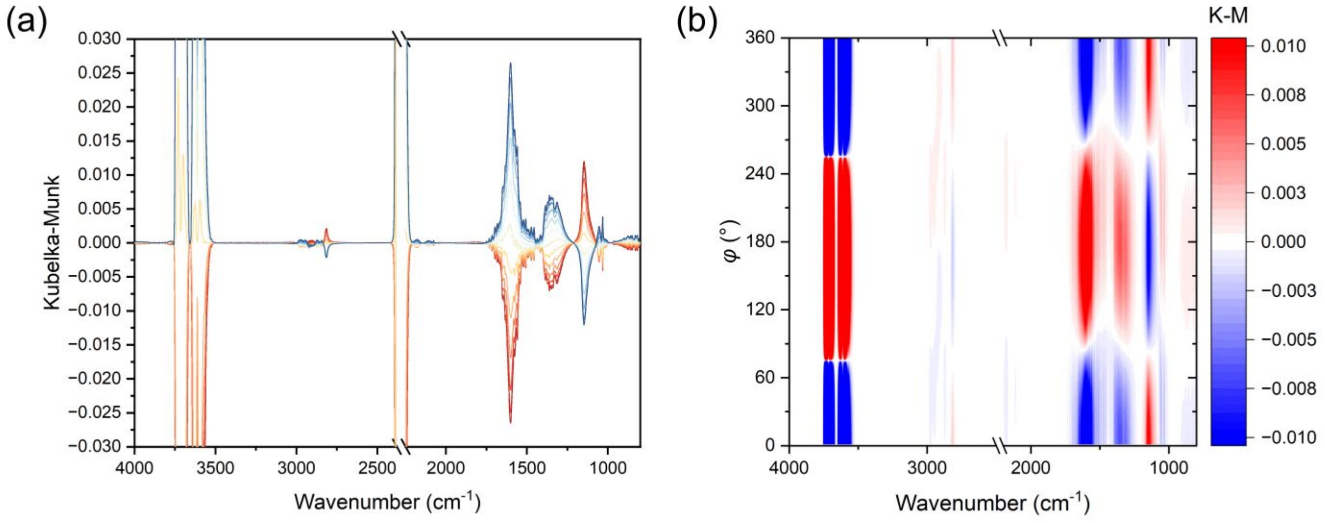

Figure S31. Phase-resolved DRIFTS spectra of CO_2 hydrogenation on 10Cu-ZrO_x at 20 bar and 260 °C.

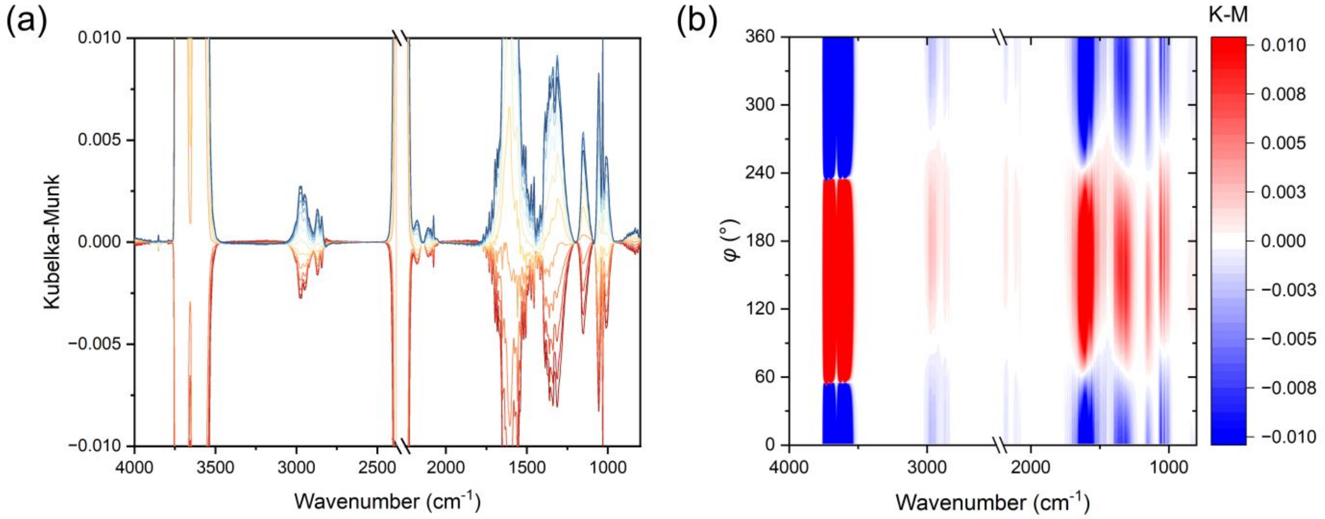

Figure S32. Phase-resolved DRIFTS spectra of CO_2 hydrogenation on 10Cu-ZrO_x at 35 bar and 260 °C.

4.6. $GaZrO_x$

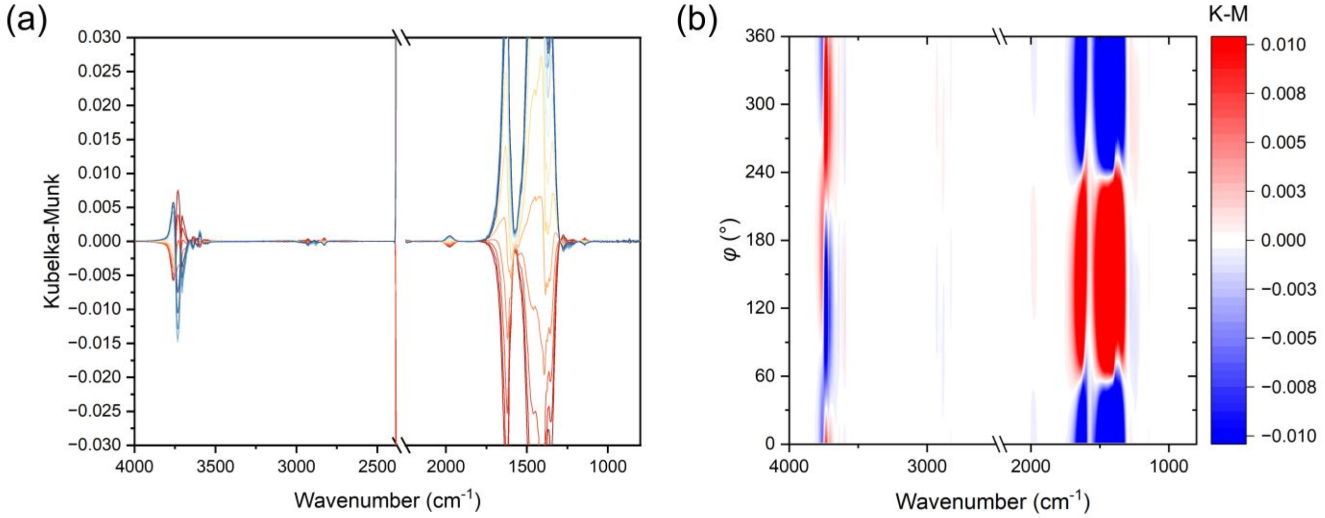

Figure S33. Phase-resolved DRIFTS spectra of CO_2 hydrogenation on $GaZrO_x$ at 1 bar and 260 °C.

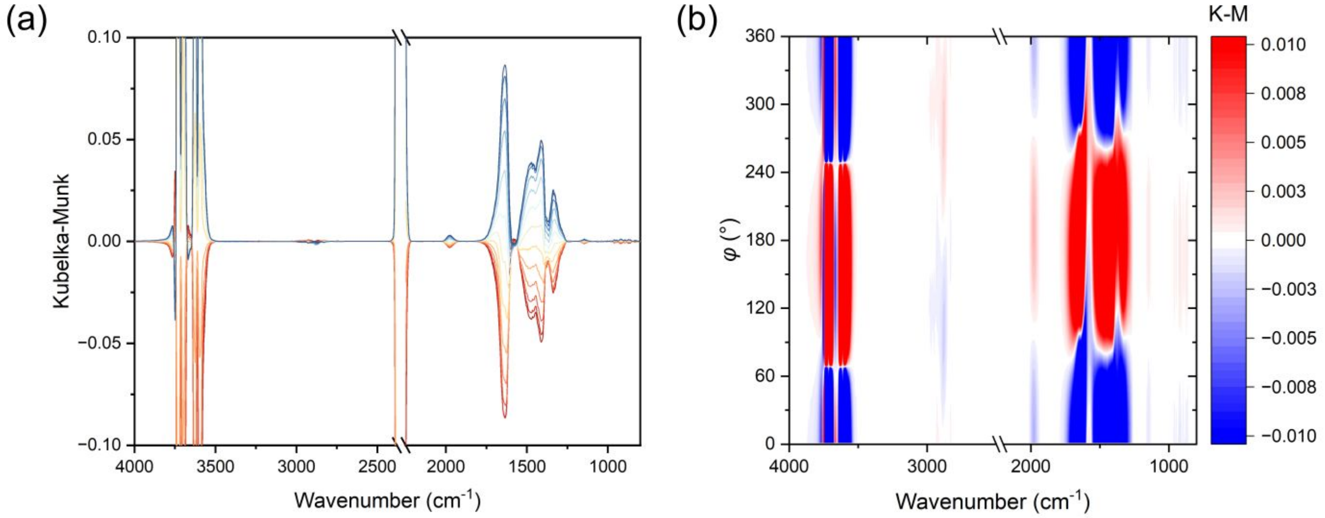

Figure S34. Phase-resolved DRIFTS spectra of CO_2 hydrogenation on $GaZrO_x$ at 20 bar and 260 °C.

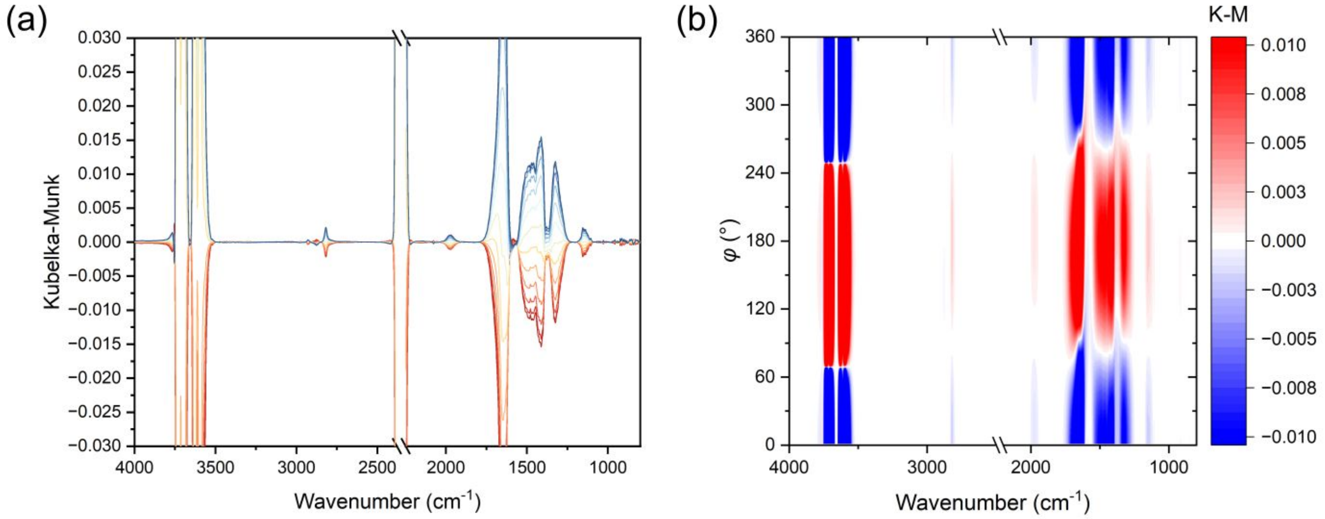

Figure S35. Phase-resolved DRIFTS spectra of CO_2 hydrogenation on $GaZrO_x$ at 20 bar and 240 °C.

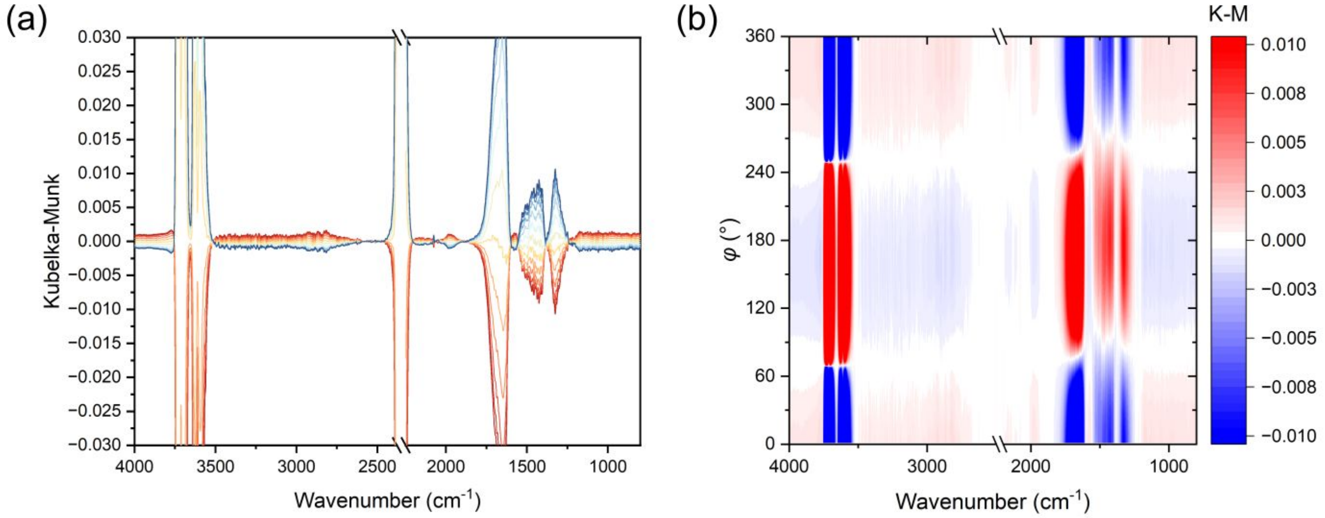

Figure S36. Phase-resolved DRIFTS spectra of CO_2 hydrogenation on $GaZrO_x$ at 20 bar and 220 °C.

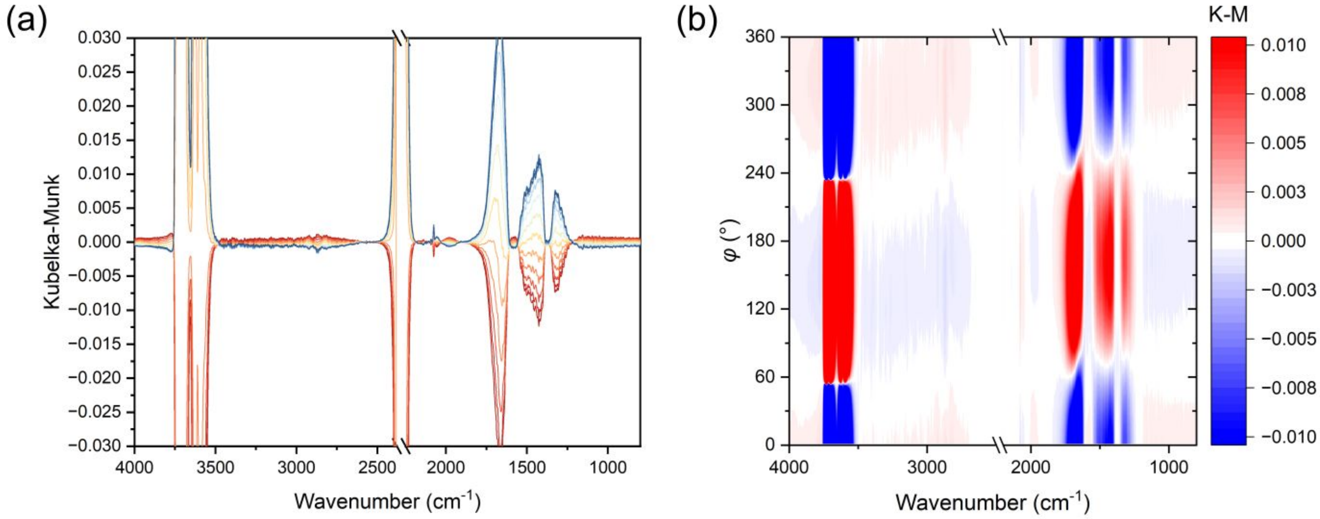

Figure S37. Phase-resolved DRIFTS spectra of CO_2 hydrogenation on $GaZrO_x$ aat 35 bar and 260 °C.

5. Supplementary figures and tables to the manuscript

Table S5. Phase delay between CO_2(g) and HCOO* during the CO_2 hydrogenation MES experiments on $CuGaZrO_x$ at the different temperatures and pressures. Only the fundamental frequency (*k* = 1) was used.

Catalyst	Pressure (bar)	Temperature (°C)	φ_{HCOO*} - $\varphi_{CO2(g)}$ (°)
10Cu-ZrO_x	1	260	10
10Cu-ZrO_x	20	260	12
10Cu-ZrO_x	35	260	12
Cu-$GaZrO_x$-48	1	260	15
Cu-$GaZrO_x$-48	20	260	13
Cu-$GaZrO_x$-48	20	240	13
Cu-$GaZrO_x$-48	20	220	13
Cu-$GaZrO_x$-48	35	260	11
Cu-$GaZrO_x$-48	50	260	15
Cu-$GaZrO_x$-24	1	260	10
Cu-$GaZrO_x$-24	20	260	10
Cu-$GaZrO_x$-24	20	240	12
Cu-$GaZrO_x$-24	20	220	17
Cu-$GaZrO_x$-24	35	260	13
Cu-$GaZrO_x$-24	50	260	16
Cu-GaO_x	1	260	8
Cu-GaO_x	20	260	24
Cu-GaO_x	20	240	23
Cu-GaO_x	20	220	33
Cu-GaO_x	35	260	23
Cu-GaO_x	50	260	20

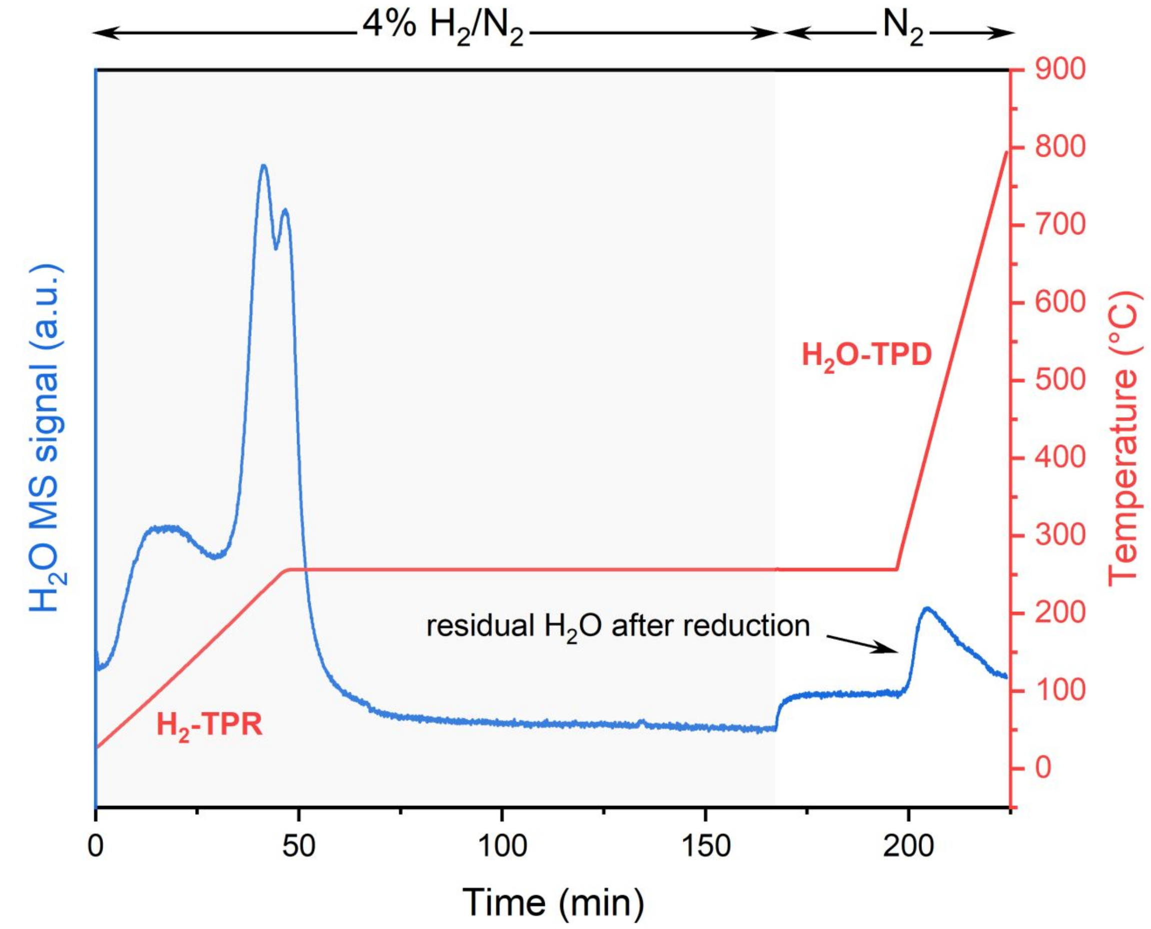

Figure S38. Water MS signal (*m/z* 18) during the temperature-programmed reduction (H_2-TPR) followed by temperature-programmed desorption of water (H_2O-TPD) experiment on Cu-GaZrO$_x$-24.

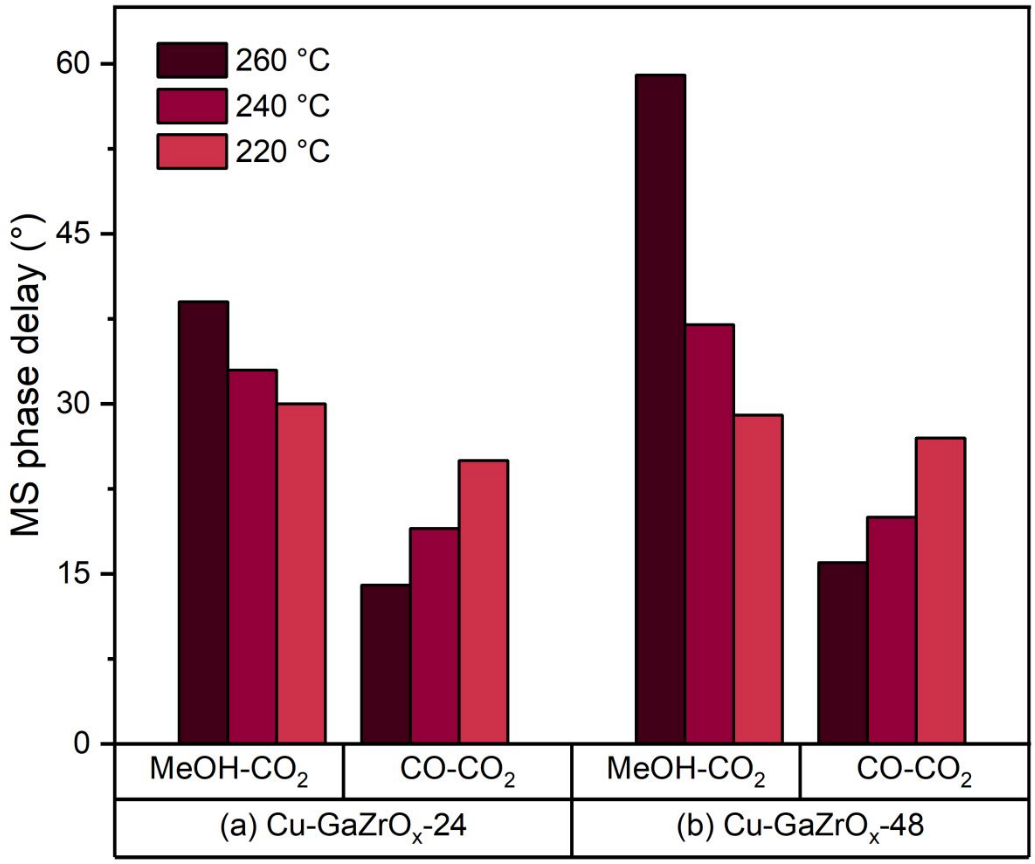

Figure S39. Effect of temperature on the MS phase delay for the formation of methanol and CO at 20 bar on (a) Cu-GaZrO$_x$-24 and (b) Cu-GaZrO$_x$-48.

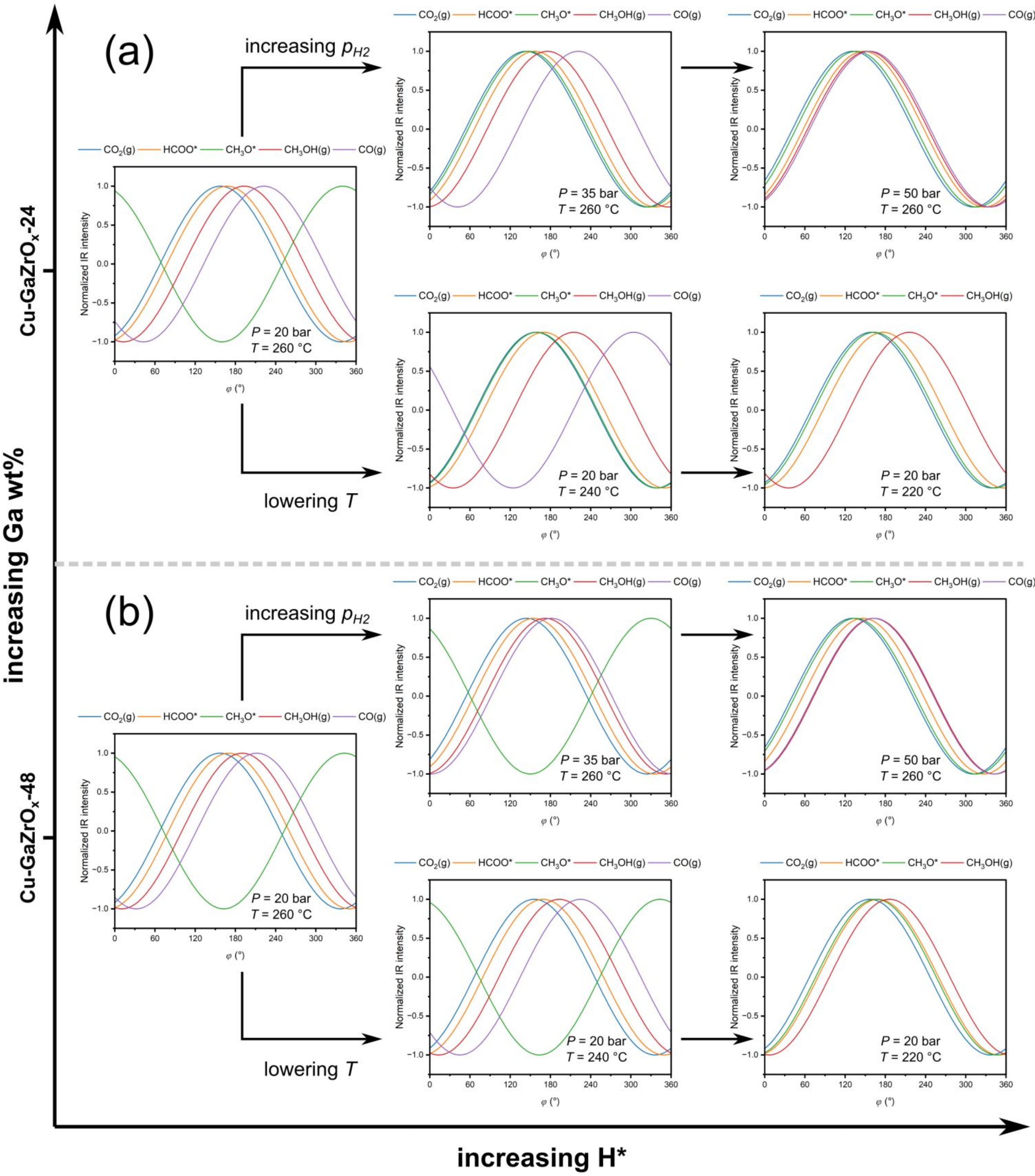

Figure S40. Normalized phase-resolved DRIFTS signal as a function of phase angle for CO_2(g), HCOO*, CH_3O*, CH_3OH(g), and CO(g) during the CO_2 hydrogenation MES experiments under varying pressure and temperature over (a) Cu-GaZrO_x-24 and (b) Cu-GaZrO_x-48. The CO(g) signals at 220 °C were omitted because of low activity.

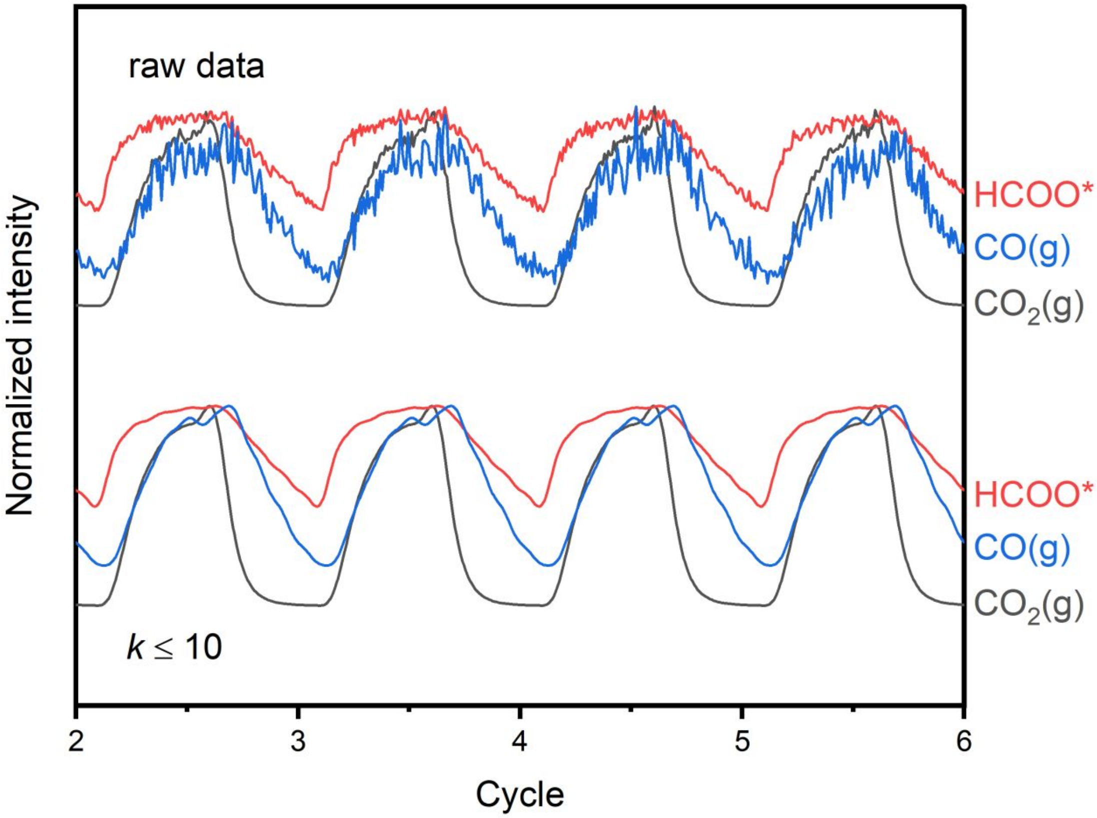

Figure S41. Normalized time-resolved DRIFTS intensities of HCOO*, CO(g), and CO_2(g) on Cu-GaZrO_x-48 at 35 bar and 260 °C. *k* refers to the demodulation index.

6. References

(1) Al Abdulghani, A. J.; Turizo-Pinilla, E. E.; Fabregas-Angulo, M. J.; Hagmann, R. H.; Ibrahim, F.; Jansen, J. H.; Agbi, T. O.; Bhat, S.; Sepúlveda-Pagán, M.; Kraimer, M. O.; Queen, C. M.; Sun, Z.; Nikolla, E.; Pagán-Torres, Y. J.; Hermans, I. Realizing Synergy between Cu, Ga, and Zr for Selective CO2 Hydrogenation to Methanol. *Appl. Catal. B Environ. Energy* **2024**, *355*, 124198. https://doi.org/10.1016/j.apcatb.2024.124198.

(2) Fehr, S. M.; Krossing, I. Spectroscopic Signatures of Pressurized Carbon Dioxide in Diffuse Reflectance Infrared Spectroscopy of Heterogeneous Catalysts. *ChemCatChem* **2020**, *12* (9), 2622–2629. https://doi.org/10.1002/cctc.201902038.

(3) Gau, A.; Hack, J.; Maeda, N.; Meier, D. M. Operando Spectroscopic Monitoring of Active Species in CO2 Hydrogenation at Elevated Pressure and Temperature: Steady-State versus Transient Analysis. *Energy & Fuels* **2021**, *35* (18), 15243–15246. https://doi.org/10.1021/acs.energyfuels.1c02592.

(4) Have, I. C. t.; Kromwijk, J. J. G.; Monai, M.; Ferri, D.; Sterk, E. B.; Meirer, F.; Weckhuysen, B. M. Uncovering the Reaction Mechanism behind CoO as Active Phase for CO2 Hydrogenation. *Nat. Commun.* **2022**, *13* (1), 324. https://doi.org/10.1038/s41467-022-27981-x.

(5) Kock, M.; Kowalewski, E.; Iltsiou, D.; Mielby, J.; Kegnæs, S. Probing the Reactive Intermediates in CO2 Hydrogenation on Ni/Al2O3 Catalysts with Modulation Excitation Spectroscopy. *ChemCatChem* **2024**, *16* (4), e202301447. https://doi.org/10.1002/cctc.202301447.

(6) Jia, H.; Feng, X.; Du, X.; Lin, L.; Mu, R.; Fu, Q. Balancing CO2 Adsorption and H2 Activation on Confined ZnOx Species for CO2 Hydrogenation. *Angew. Chem. Int. Ed.* **2025**, *64* (25), e202503319. https://doi.org/10.1002/anie.202503319.

(7) Dostagir, N. H. M. D.; Tomuschat, C. R.; Oshiro, K.; Gao, M.; Hasegawa, J.; Fukuoka, A.; Shrotri, A. Mitigating the Poisoning Effect of Formate during CO2 Hydrogenation to Methanol over Co-Containing Dual-Atom Oxide Catalysts. *JACS Au* **2024**, *4* (3), 1048–1058. https://doi.org/10.1021/jacsau.3c00789.

(8) Frigo, M.; Johnson, S. G. The Design and Implementation of FFTW3. *Proc. IEEE* **2005**, *93* (2), 216–231. https://doi.org/10.1109/JPROC.2004.840301.

(9) Calatayud, M.; Collins, S. E.; Baltanás, M. A.; Bonivardi, A. L. Stability of Formate Species on β-Ga2O3. *Phys. Chem. Chem. Phys.* **2009**, *11* (9), 1397–1405. https://doi.org/10.1039/B800519B.

(10) Ma, Z.-Y.; Yang, C.; Wei, W.; Li, W.-H.; Sun, Y.-H. Surface Properties and CO Adsorption on Zirconia Polymorphs. *J. Mol. Catal. A Chem.* **2005**, *227* (1), 119–124. https://doi.org/10.1016/j.molcata.2004.10.017.

(11) Bensitel, M.; Moraver, V.; Lamotte, J.; Saur, O.; Lavalley, J.-C. Infrared Study of Alcohols Adsorption on Zirconium Oxide: Reactivity of Alkoxy Species towards CO2. *Spectrochim. Acta A Mol. Spectrosc.* **1987**, *43* (12), 1487–1491. https://doi.org/10.1016/S0584-8539(87)80035-1.
